# Ceramide and the membrane-fusion activity of LC3/GABARAP autophagy proteins

**DOI:** 10.1007/s00018-025-05811-9

**Published:** 2025-07-19

**Authors:** Yaiza R. Varela, Camila C. Aguirre, Marina N. Iriondo, Uxue Ballesteros, M. Isabel Collado, Asier Etxaniz, L. Ruth Montes, Felix M. Goñi, Alicia Alonso

**Affiliations:** 1 Department of Biochemistry and Molecular Biology, Instituto Biofisika (UPV/EHU, CSIC), University of the Basque Country, Leioa, E-48940 Spain; 2 SGIKER, Universidad del País Vasco, Barrio Sarriena s/n, Leioa, 48940 Spain; 3 Donostia International Physics Center (DIPC), Paseo Manuel de Lardizabal 4, Donostia-San Sebastián, 20018 Spain

**Keywords:** Ceramide, LC3/GABARAP, Membrane fusion, Autophagy, Autophagosome

## Abstract

Macroautophagy is a cellular degradation process characterized by the formation of the double-membrane structure termed autophagosome (AP). The process of AP formation is not fully understood, but it is thought to happen through the combined action of direct lipid transfer and incorporation of new vesicles to the edges of the growing structure. Human LC3/GABARAP autophagy-related proteins are known to induce vesicle tethering and lipid mixing in vitro, which makes them suitable for the latter expansion mechanism. Ceramide (Cer) is a sphingolipid previously described to facilitate membrane fusion. Cer has also been related to macroautophagy modulation previously, although its specific role remains unclear. Moreover, the presence of sphingolipids in the AP has been suggested by recent experiments, increasing the relevance of Cer in macroautophagy. The present work has investigated the potential role that Cer could have on the proposed fusion of new vesicles to the nascent AP membrane. Interaction of purified ATG proteins with lipid vesicles of defined composition has been quantified using fluorescence spectroscopic techniques. Our results suggest that, if present, Cer could promote the vesicle tethering and leakage-free intervesicular lipid mixing induced by GABARAP and GABARAPL1, which would in turn mediate AP membrane expansion.

**Supplementary Information:**

The online version contains supplementary material available at 10.1007/s00018-025-05811-9.

## Introduction

Autophagy is a cytoplasmic degradation process, mainly of macromolecules and organelles, which has been evolutionarily conserved and is key to ensure cellular and tissue homeostasis [1]. One of the most studied autophagy types is macroautophagy (hereafter autophagy), which requires the formation of a special organelle called autophagosome (AP), limited by a double membrane. AP formation originates from the expansion of an initial cistern-like structure (phagophore), which eventually closes giving rise to the double-membrane AP [2–6].

For this purpose, additional lipids must be transferred to or incorporated into the phagophore. Different mechanisms have been described for this event, involving the participation of autophagy-related proteins (ATG) that mediate direct lipid transfer [7] or incorporation of new vesicles. The present work deals with the latter process, which involves the addition of vesicle lipids to the edge of the cup-shaped phagophore.

The LC3/GABARAP protein family is formed by the six human orthologs of yeast Atg8. Regarding phagophore growth, the LC3/GABARAP proteins could mediate vesicle incorporation, as they are known to induce in vitro vesicle tethering and intervesicular lipid mixing [8–11]. In this process, LC3/GABARAP proteins are first activated by exposure of a C-terminal Gly. This residue allows the covalent protein anchoring to the membrane lipid phosphatidylethanolamine (PE) through the coordinated action of two ubiquitin-like systems, namely the ATG12 and the LC3/GABARAP systems [5, 12]. The ATG12 ubiquitin-like system is required for in vivo formation of the ATG12–ATG5-ATG16L1 complex, however, the latter complex is not essential for the in vitro lipidation of LC3/GABARAP [8, 9].

AP lipid composition is not completely understood, however, recent data by Schmitt et al. [13] suggested that sphingolipids (SL) (mainly, but not exclusively sphingomyelin, SM) would make up to 20 mol% of the AP lipids. SL could be incorporated into the phagophore membrane through either of the two proposed expansion systems. Direct lipid transfer is thought to happen mainly from the ER, where ceramide (Cer) is synthesized. LC3/GABARAP proteins could also incorporate lipids from SL-containing donor membranes or vesicles. Sentelle et al. [14] showed the involvement of Cer in autophagy, although additional results suggested that there is no direct interaction between the soluble proteins and Cer [15]. Moreover, the Cer potential role in LC3/GABARAP-membrane interactions has been explored [16] and its role in physiological processes such as autophagy has been demonstrated [17].

In order to assess the influence of Cer on the LC3/GABARAP-induced extensive vesicle fusion required for phagophore expansion, a combination of experiments was designed. This sphingolipid has been the focus of this work, as its structural properties are known to favour fusion [17, 18]. First, the in vitro reconstitution of PE-binding (lipidation) was assessed, in the presence of ATG7, ATG3 and ATP [9]. Then, vesicle tethering or aggregation induced by the lipidated proteins, this being the first step required for vesicle-vesicle fusion, was considered. Next, intervesicular total lipid mixing (TLM) was used to analyze the protein fusogenic capacity. Separate assays for intervesicular mixing of inner-monolayer lipids (ILM) and for intervesicular aqueous contents mixing (ACM) were used to discern whether the proteins were inducing full fusion or hemifusion events [19–21]. These studies were carried out with membrane compositions that either lacked or contained Cer (PC: PE vs. PC: PE: Cer). The results support a clear enhancing effect of Cer in LC3/GABARAP-induced vesicle membrane fusion.

## Materials and methods

### Materials

L-α-phosphatidylcholine from hen egg yolk (ePC, 840051), 1,2-dioleoyl*-sn-*glycero-3-phosphatidylethanolamine (DOPE, 850725), 1,2-dioleoyl*-sn-*glycero-3-phosphoethanolamine-N-[4-(p-maleimidomethyl) cyclohexane-carboxamide] (PEmal, 780201), ceramide (egg, chicken) (eCer, 860051), 1,2-dioleoyl*-sn-*glycero-3-phosphatidylethanolamine-N-(lissamine rhodamine B sulfonyl) (Rho-PE, 810150), were purchased from Avanti Polar Lipids, Inc. (Alabaster, AL). N-(7-nitrobenz-2-oxa-1,3-diazol-4-yl)−1,2-dihexadecanoyl*-sn-*glycero-3-phosphatidylethanolamine (NBD-PE, N360), p-xylene-bis-pyridinium bromide (DPX, X-1525) and 8-aminonaphthalene-1,3,6-trisulfonic acid, disodium salt (ANTS, A350) were purchased from Thermo Fisher Scientific (Waltham, MA). Methanol and chloroform were from Fisher (Suwanee, GA). Buffer solution, unless otherwise stated, was System Buffer: 50 mM Tris-HCl, 150 mM NaCl, pH 7.4. All salts and organic solvents were of analytical grade.

### DNA constructs

The pGEX4T-1 plasmids for the expression of six glutathione S-transferase (GST)-tagged human orthologs of yeast Atg8, (human LC3A, human LC3B, human LC3C, human GABARAP, human GABARAPL1 and human GABARAPL2) were kindly provided by Dr. Ivanna Novak (School of Medicine, University of Split, Croatia). Each of these proteins was in a truncated form, lacking the C-terminal glycine. The glycine-exposed forms used in this work were constructed using a QuikChange site-directed mutagenesis kit (200514, Stratagene, San Diego, CA). To obtain Cys-terminal proteins, the exposed Gly was mutated to a Cys through site-directed mutagenesis by GenScript (Leiden, Netherlands). The pGEX6P-1 plasmid for expression of human ATG3 was kindly provided by Dr. Isei Tanida (National Institute of Infectious Diseases, Tokyo, Japan). The pFast BacHT(B) plasmid for expression of mouse ATG7 was kindly supplied by Dr. S. Martens (Max Perutz Labs, Vienna, Austria).

### Recombinant protein expression and purification

LC3/GABARAP proteins were purified from soluble fractions of bacterial extracts obtained in the absence of detergents, and they were > 90% pure as evaluated by Coomassie Brilliant Blue-stained SDS-PAGE. *Escherichia coli* BL21 (λDE3) cells were transformed with the appropriate plasmids. After breaking the cells by sonication and removing cellular debris by centrifugation at 30,000 x g for 30 min at 4 °C, the sample supernatant fraction was incubated with 1 mL Glutathione Sepharose 4B beads for 3 h at 4 °C to bind GST-tagged proteins. Bound proteins were cleaved with Thrombin Protease (GE Healthcare, 27–0846-01) overnight at room temperature in Thrombin Cleavage Buffer (140 mM NaCl, 2.7 mM KCl, 10 mM Na_2_HPO_4_, 1.8 mM KH_2_PO_4_, 1 mM DTT, pH 7.3). ATG3 protein was cleaved with PreScission Protease (GE Healthcare, 27-0843-01) for 4 h at 4 °C in PreScission Cleavage Buffer (50 mM Tris-HCl, 150 mM NaCl, 1 mM EDTA, pH 7.5). After cleavage, fractions were eluted, concentrated to 500 µL and loaded onto a Superdex 75 10/300 GL size exclusion column equilibrated in 50 mM Tris-HCl, 150 mM NaCl, 1 mM EDTA, 1 mM DTT, pH 7.5 buffer. Proteins were then aliquoted, flash-frozen and stored in 20% glycerol at −80 °C until further use.

Mouse ATG7 (mATG7) was expressed in Sf9 insect cells that were then harvested (see details in [9]) and kept frozen until use. It was purified in the absence of detergents and > 90% purity was achieved as evaluated by Coomassie Brilliant Blue-stained SDS-PAGE. For purification, pellets were thawed and resuspended in ice-cold breaking buffer [50 mM HEPES, 300 mM NaCl, 10 mM imidazole, 2 mM Tris (2-carboxyethyl) phosphine (TCEP) pH 7.5, supplemented with complete protease inhibitors (11836170001, Sigma), protease inhibitor cocktail (P8849, Sigma), and 1 µL benzonase nuclease (E1014, Sigma)]. Cells were lysed on ice by extrusion in a tissue homogenizer, and lysates were cleared by centrifugation. Supernatant was applied to a 5-mL nickel-nitrilotriacetic acid (Ni–NTA) column and eluted via a stepwise imidazole gradient (50, 75, 100, 150, 200, and 300 mM). Fractions containing the mATG7 were pooled, concentrated, applied onto a Superdex 200 10/300 GL column, and eluted in 25 mM HEPES, 150 mM NaCl, 1 mM DTT, pH 7.5 buffer. Fractions containing pure mATG7 were pooled, concentrated, flash-frozen in liquid nitrogen, and stored at −80 °C.

### Large unilamellar vesicle (LUV) preparation

The appropriate lipids were mixed in organic solution and the solvent was evaporated to dryness under a N_2_ stream. Then, the sample was kept under high vacuum for 1 h to remove solvent traces. The lipids were swollen in System Buffer in order to obtain multilamellar lipid vesicles (MLV). When required, hydration was enhanced by stirring with a glass rod, and the vesicles were homogenized by forcing the sample ≈ 80 times between two syringes through a narrow tube (0.5-mm internal diameter, 10-cm long) at 50 °C. MLV were subjected to 10 freeze/thaw cycles, and then extruded using 0.05-µm (110,603 Whatman) or 0.1-µm (110,605) pore size Nuclepore filters to obtain ≈ 80-nm or ≈ 100-nm LUV, respectively [22]. LUV were centrifuged for 30 min at 9,000 x g and 4 °C, and the supernatants were saved for further use. Vesicle size was checked using a Malvern Zeta-Sizer Nano ZS (Malvern, Instruments, UK). Phospholipid concentration was determined with a phosphate assay [23].

### Cer quantitation by thin layer chromatography (TLC)

The lipid mixtures were dried under high vacuum, resuspended in chloroform and kept at −20 °C until loading them on the TLC plate. LUV were obtained as described above, and a lipid extraction was performed by adding 800 µL LUV to 4.2 mL chloroform: methanol (2:1), obtaining a mixture of chloroform: methanol: H_2_O (2:1:0.6 by vol.). After vortexing, the mixture was centrifuged for 20 min at 1,700 x g and 4 °C [20]. The bottom (organic) phase, containing the lipids, was recovered and kept at −20 °C. When needed, the organic solvent was dried under hIgh vacuum and resuspended in chloroform to load it on the TLC plate.

TLC Silica gel 60 plates (1.05553.0001, Merck) were activated by soaking them in a solution containing 1% (g/mL) sodium oxalate and 2 mM EDTA at room temperature for 30 min. The plates were allowed to dry for 15 min at room temperature, then at 100 °C for 1 h. The composition of the mobile phase was: chloroform (46%), acetone (17.24%), methanol (14.4%), acetic acid glacial (13.8%), distilled water (8%). After running the TLC, the plates were immersed in 5% (v/v) H_2_SO_4_, then heated for 5 min at 110 °C in order to detect the lipidic components. Spot intensities were quantified [GS-800 Calibrated Densitometer (Bio-Rad, California, USA), ImageJ] by comparison with authentic internal standards.

### Chemical and enzymatic lipidation assays

Autophagy activation requires lipidation of LC3/GABARAP, i.e. their covalent binding to PE. In vitro lipidation can be induced either using all the proteins involved in the cell reaction (enzymatic method), or with the chemical method, which requires modified proteins and lipids. The LC3/GABARAP–PE covalent complex is not broken down during sample preparation and the lipidated proteins migrate faster than their non-lipidated counterparts in SDS-PAGE gels [24]. Cells keep a basal level of autophagy to maintain the correct turnover of macromolecules, and the ratio between the membrane-bound and soluble forms of LC3/GABARAP proteins is usually taken as an indicator of autophagy levels and autophagy activation in cells. With liposome systems and purified LC3/GABARAP, this method is also useful to detect LC3/GABARAP–PE interaction [9, 11].

For the chemical lipidation assay, proteins were modified to contain a C-terminal Cys. The purified proteins (5 µM) were incubated with 30 mol% PEmal-containing liposomes at a total final lipid concentration of 0.4 mM in System Buffer. PEmal was a partial substitute for DOPE. The mixture was incubated at 37 °C in System Buffer under continuous shaking (1,100 rpm).

For the enzymatic lipidation approach, purified ATG7 (0.5 µM), ATG3 (2 µM), MgCl_2_ (1 mM), and the pertinent Gly-terminal LC3/GABARAP protein (5 µM) were mixed with 0.4 mM liposomes in System Buffer. Reactions were performed at 37 °C and initiated by addition of 5 mM ATP.

In both approaches, 30 µL of the reaction mixture were sampled at different time points, mixed with 6 µL of 6x Protein Loading dye and heated at 100 °C for 5 min. Lipidation in each sample was analyzed in SDS-PAGE gels using Comassie Brilliant Blue staining. The gels of three independent experiments were quantified using Image J. The amounts of PE-bound and -unbound LC3/GABARAP at each time point were measured as the areas below the corresponding intensity peaks. The % PE-bound LC3/GABARAP relative to total protein (% lipidation) was computed for each time point and plotted as a function of time.

### Vesicle tethering assays

Liposome tethering/aggregation was monitored in a Varian Cary 300 (Agilent Technologies, Santa Clara, CA) spectrophotometer as an increase in turbidity (absorbance at 400 nm) of the sample [8]. LUV were prepared in System Buffer (see Materials) and all assays were carried out at 37 °C with continuous stirring. The reaction was started by adding protein or ATP as required.

### Vesicle total and inner-monolayer lipid mixing assays

A Förster resonance energy transfer assay was used to monitor intervesicular lipid mixing. For total lipid mixing (TLM), LUV were prepared in System Buffer (see Materials). Then, the appropriate LUV, containing 1.5 mol% Rho-PE and 1.5 mol% NBD-PE (labeled in the head group), were mixed with a 9-fold excess unlabeled liposomes [25]. To start the reaction, ATP was added to the mixture after 5 min. NBD emission was monitored setting the excitation and emission wavelengths at 465 nm and 530 nm respectively (excitation and emission maxima) in a Fluorolog^®^−3 (Horiba Jobin Yvon, Edison, NJ, USA) spectrofluorometer, with the slits at 2 nm, and placing a 515 nm cut-off filter before the emission monochromator. Inner-monolayer lipid mixing (ILM) was measured using asymmetrically labeled lipid vesicles. The latter were obtained by quenching the outer leaflet NBD-PE fluorescence, upon addition of sodium dithionite [26]. Excess sodium dithionite was removed by gel filtration in Sephadex G-25 M, using System Buffer for elution. 100% intervesicular total lipid mixing and 100% inner-monolayer lipid mixing were established by adding Triton X-100 to a 0.25% (v/v) final concentration. The extent of either total or inner-monolayer lipid mixing was computed with the equation: % LM = (F_t_-F_0_)/(F_100_-F_0_) x 100, where LM is lipid mixing, either total or inner-monolayer, F_t_ is the NBD-PE fluorescence of LUV at time t, F_0_ is the NBD-PE fluorescence of LUV before ATP addition (when the NBD-Rho energy transfer is maximum), and F_100_ is the maximum NBD-PE fluorescence value after LUV disruption by addition of Triton X-100.

### Vesicle aqueous contents leakage assay

Leakage of vesicle aqueous contents was monitored with the ANTS/DPX leakage assay [27]. LUV were prepared in ANTS/DPX buffer (20 mM ANTS, 70 mM DPX, 50 mM Tris-HCl, 40 mM NaCl, pH 7.5). Non-encapsulated ANTS and DPX were removed by gel filtration in Sephadex G-25 M, using System Buffer for elution. ANTS emission was monitored at 520 nm with the excitation wavelength set at 355 nm. ATP was added to the mixture after 5 min, to start the reaction. To establish the 100% leakage signal, Triton X-100 was added to a 0.25% (v/v) final concentration. The extent of leakage was quantified with the equation: % Leakage = (F_t_-F_o_)/(F_100_-F_0_) x 100, where F_t_ is the ANTS fluorescence of LUV at time t, F_0_ is the ANTS fluorescence of LUV before ATP addition, and F_100_ is the maximum ANTS fluorescence value after disruption of LUV by addition of Triton X-100.

### Vesicle aqueous contents mixing assay

Intervesicular aqueous contents mixing (ACM) was monitored using the ANTS/DPX mixing assay [27]. In this case, three LUV populations of each lipid composition were prepared in three different buffers, either containing only ANTS (39 mM ANTS, 50 mM Tris-HCl, 72 mM NaCl, pH 7.5), only DPX (140 mM DPX, 50 mM Tris-HCl, 10 mM NaCl, pH 7.5) or both (20 mM ANTS, 70 mM DPX, 50 mM Tris-HCl, 40 mM NaCl, pH 7.5). Non-encapsulated ANTS and/or DPX were removed by gel filtration in Sephadex G-25 M, using isosmotic System Buffer for elution. The osmolarity of all the buffers was checked in an Osmomat 030 (Berlin, Germany) osmometer to ensure that all buffers were isotonic. ANTS emission was monitored at 520 nm. ATP was added to the mixture after 5 min. 0% vesicle contents mixing was set by using a 1:1 mixture of ANTS- and DPX-containing liposomes. 100% contents mixing corresponded to the fluorescence of the vesicles containing co-encapsulated ANTS and DPX. The extent of aqueous contents mixing was quantified on a percentage basis according to the equation: % ACM= (-(F_t_-F_o_)/(F_100_-F_0_)) x 100, where F_t_ is the ANTS fluorescence of LUV at time t, F_0_ is the ANTS fluorescence of LUV before ATP addition, and F_100_ is the ANTS/DPX liposome fluorescence value, i.e. the minimum ANTS fluorescence value that can be obtained.

### Phosphorus 31-NMR (^31^P-NMR)

60 mM lipid in the form of multilamellar vesicles (MLV) was transferred to 5 mm NMR tubes. Data acquisition was performed in a Bruker AV500 spectrometer (Rheinstetten, Germany) operating at 202.45 MHz for ^31^P with a 5-mm BBI probe with z-axis gradient, at 37 °C, or at 50 °C. The data were recorded and processed with software TOPSPIN 2.1 (Bruker).

### Statistics

Unless otherwise stated, all data are given as average values ± SD of three measurements obtained with different vesicle preparations. When required, statistical significance was measured with the Student’s t-test.

## Results

### GABARAP and GABARAPL1 showed the highest membrane tethering ability among the six homologs tested

In order to study the effect of Cer on LC3/GABARAP-induced vesicle tethering, the first step was to reconstitute protein lipidation (PE binding) with liposomes either lacking or containing Cer. Egg ceramide (eCer), consisting mainly of C16 Cer, was used as the standard Cer in our experiments. A high (50 mol %) PE concentration was used to favor the covalent binding of LC3/GABARAP to the liposomes. The liposome compositions used in our comparative studies were PC: PE (50:50) and PC: PE: Cer (40:50:10). Lipid compositions are given as mol ratios in all cases. Two different lipidation methods were used in order to test the potential role of Cer in LC3/GABARAP-induced vesicle tethering, namely the chemical and the enzymatic procedures.

#### Chemical lipidation approach

The chemical lipidation method was used as an initial approach to test the vesicle-tethering capacity of the six homologs. This method had been previously used and its simple design allowed exploring the behavior of the six LC3/GABARAP proteins [8, 11].

The chemical lipidation approach requires the reaction between a maleimide group and a Cys amino acid residue. With this purpose, the LC3/GABARAP proteins were modified so that they exposed a C-terminal Cys, instead of the native Gly. The following mutants were thus obtained: LC3A G120C, LC3B G120C, LC3C G126C, GABARAP G116C, GABARAPL1 G116C and GABARAPL2 G116C. As homologs LC3A and GABARAPL2 possess an additional (native) Cys residue in their sequence, the native Cys in those proteins was mutated to Ser in order to avoid unwanted Cys-maleimide interactions. Thus, LC3A C17S G120C and GABARAPL2 C15S G116C proteins were obtained. In addition, 60 mol% of the PE present in the vesicles was modified through binding a maleimide group (PEmal). This allows the protein C-terminal Cys to react with the maleimide moiety, and anchor the membrane. The compositions tested with the chemical approach were ePC: DOPE: PEmal (50:20:30) and ePC: DOPE: PEmal: eCer (40:20:30:10).

**Fig. 1:**
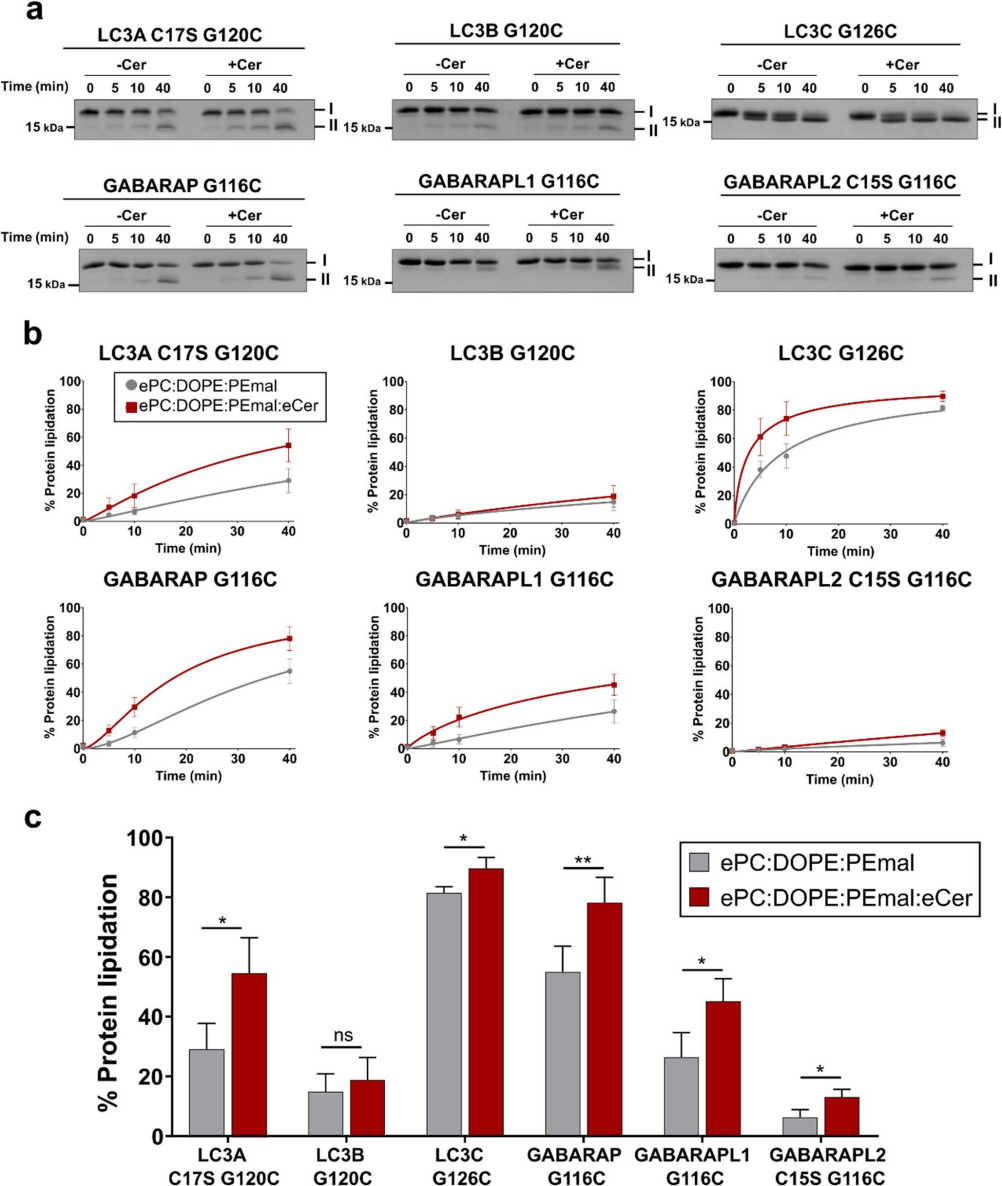
Ceramide enhances chemical lipidation of LC3/GABARAP proteins. Protein Cys-terminal lipidation measured at times 0, 5, 10, and 40 min. (**a**) SDS-PAGE gels representative for each protein and lipid composition. **I**, non-lipidated protein; **II**, lipidated protein. (**b**) Lipidation time courses. (**c**) Lipidation at time 40 min. 0.4 mM LUV composed of ePC: DOPE: PEmal (50:20:30) (gray) or ePC: DOPE: PEmal: eCer (40:20:30:10) (red) were mixed with 5 µM of the pertinent LC3/GABARAP protein. Liposomes were ≈ 100 nm in diameter. Average data ± S.D., *n* = 3. **p < 0.01, *p < 0.05, ns: non-significant differences

First, LC3/GABARAP protein lipidation was assayed (Fig. 1) to discern the Cer effect on the PE interaction with each of the six homologs. Proteins and liposomes were incubated at 37 °C and aliquots were taken for each protein at different times (0, 5, 10, 40 min).

The percent PE-bound (lipidated) LC3/GABARAP can be estimated by densitometry as it migrates faster than the unbound protein in SDS-PAGE gels. In Fig. 1a, I and **II** denote respectively the non-lipidated and lipidated forms of the homologs. The percent lipidated protein in Fig. 1b, c is computed from the densitometric intensities of the bands.

All the proteins, except LC3B G120C and GABARAPL2 C15S G120C, showed a Cer-enhanced initial lipidation rate (Fig. 1b). In the absence of Cer, LC3A C17S G120C, GABARAP G116C and GABARAPL1 G116C showed lower lipidation levels than LC3C G126C (Fig. 1c, gray bars). In fact, LC3C G126C was the homolog reaching highest lipidation rates (Fig. 1b, LC3C G126C panel) and top levels after 40 min (Fig. 1c), > 80% for both lipid compositions. Note that the basal lipidation levels were above 30% in those four cases. In addition, a significant protein lipidation increase was observed in the presence of Cer (Fig. 1c, red bars), particularly for GABARAP G116C (Fig. 1c). LC3B G120C and GABARAPL2 C15S G116C were the homologs reaching lowest lipidation levels, both below 30%, with either lipid composition (Fig. 1b, c). In summary, protein lipidation was clearly improved by Cer whenever Cer-free lipidation was above ≈ 30%.

The possible Cer dose-dependence of LC3/GABARAP lipidation was tested with LC3A and GABARAPL1, using LUV composed of ePC: DOPE: PEmal: Cer (50-x:20:30:x), in which the mol % Cer concentration was x = 0, 5, 10 or 15, higher concentrations leading to very unstable bilayers [28]. The results are shown in Supplementary Fig. 1. Only a slight correlation was seen, lipidation increasing but moderately with Cer concentration. Our interpretation of the data is based on the immiscibility of Cer with ePC or PE [18, 28]. Cer tends to separate into rigid domains, and the concentration of “active” monomeric (or oligomeric) Cer in the fluid bilayers is virtually constant above 5 mol % [17, 29].

**Fig. 2:**
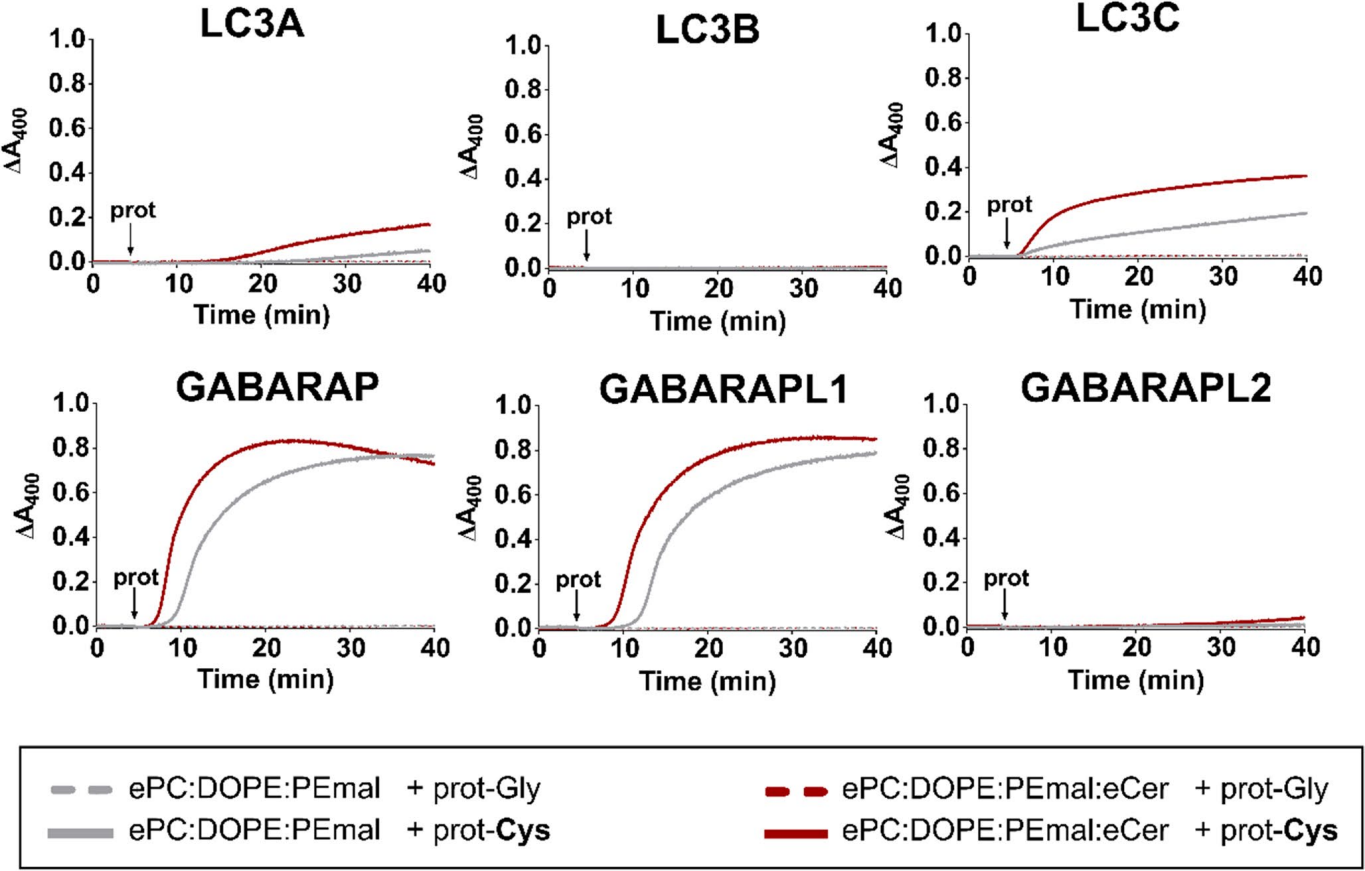
Ceramide enhances vesicle tethering promoted by chemically lipidated LC3/GABARAP proteins. Liposome-tethering activity assayed as an increase in absorbance at 400 nm (ΔA_400_). 0.4 mM LUV composed of ePC: DOPE: PEmal (50:20:30) (gray) or ePC: DOPE: PEmal: eCer (40:20:30:10) (red) were mixed with 5 µM of the pertinent LC3/GABARAP protein. The arrows indicate protein addition. Solid lines, Cys-terminal proteins: LC3A C17S G120C, LC3B G120C, LC3C G126C, GABARAP G116C, GABARAPL1 G116C and GABARAPL2 C15S G116C. Dashed lines, *control* experiments, Gly-terminal proteins: LC3A C17S, LC3B, LC3C, GABARAP, GABARAPL1 and GABARAPL2 C15S. Liposomes were ≈ 100 nm in diameter. Average data ± S.D., *n* = 3, *n* = 2 for the dashed *control* curves. The dashed lines are often indistinguishable from the X axis

Next, in order to study whether a Cer effect was also noticeable on the LC3/GABARAP-promoted vesicle tethering, liposomes of the desired lipid compositions were diluted in buffer and suspension turbidity measured as absorbance at 400 nm (A_400_). Once a stable baseline was observed for 5 min, the Cys-terminal LC3/GABARAP protein was added (Fig. 2). The Gly-terminal counterparts of the proteins were used as controls (Fig. 2, dashed lines)

Experiments in Fig. 2 show that those proteins that reached lipidation levels below 30% **(**Fig. 1c**)**, LC3B G120C and GABARAPL2 C15S G116C, were unable to induce vesicle tethering even 35 min after protein addition (Fig. 2, LC3B and GABARAPL2 panels). These results were indistinguishable from those with the corresponding Gly-terminal variants of the latter proteins, which could not be chemically lipidated (Fig. 2, dashed lines).

The other four proteins (LC3A C17S G120C, LC3C G126C, GABARAP G116C and GABARAPL1 G116C) caused liposome tethering only when the Cys-terminal forms were used, underlining the requirement of lipidation for vesicle tethering (Fig. 2, solid lines). GABARAP and GABARAPL1 were by far the two most efficient proteins in terms of vesicle tethering (Fig. 2). Furthermore, those four proteins showed higher tethering activities when Cer was part of the lipid composition (Fig. 2, solid red lines vs. solid gray lines).

**Fig. 3:**
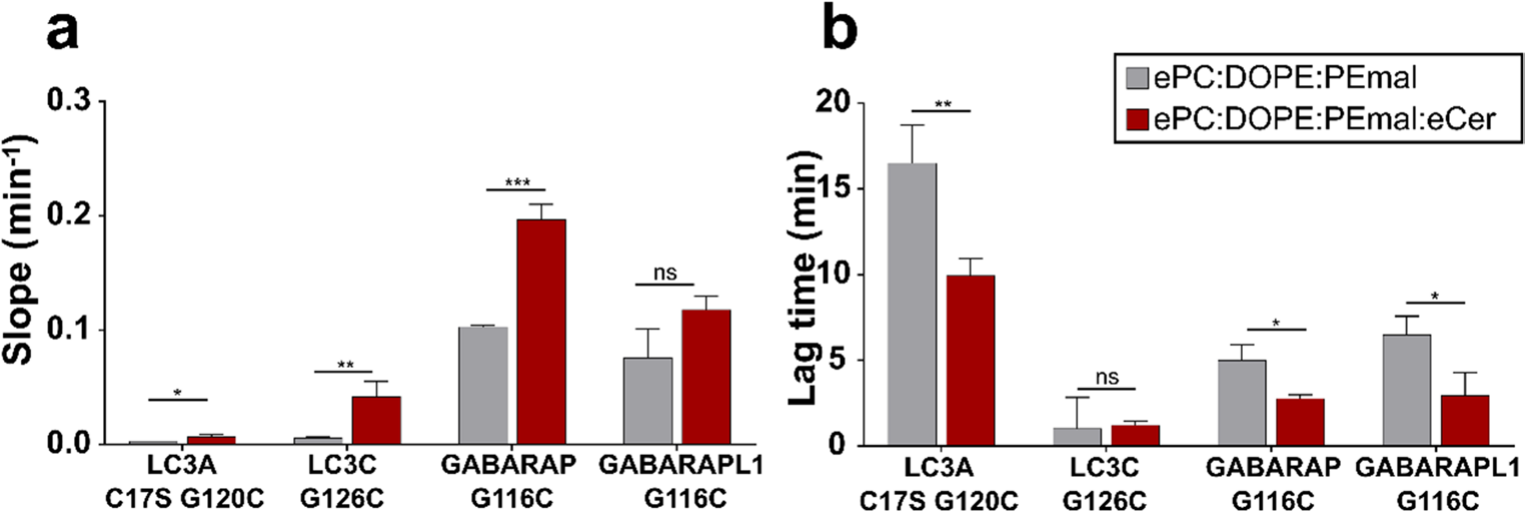
Ceramide increases rates and shortens lag times of vesicle tethering induced by chemically lipidated LC3/GABARAP. (**a**) Vesicle tethering rates. (**b**) Lag times. Data obtained with proteins susceptible to lipidation, as in Fig. 2. 0.4 mM LUV composed of ePC: DOPE: PEmal (50:20:30) (gray bars) or ePC: DOPE: PEmal: eCer (40:20:30:10) (red bars) were mixed with 5 µM protein. Liposomes were ≈ 100 nm in diameter. Representative curves are shown in Fig. 2. Average data ± S.D., *n* = 3. ***p < 0.001, **p < 0.01, *p < 0.05, ns: non-significant differences

From the results in Fig. 2, two parameters were computed, namely the maximum initial slope (Fig. 3a) (corresponding to the vesicle tethering rate) and the lag times (Fig. 3b), measured from the time of protein addition. They were not computed for LC3B G120C or GABARAPL2 C15S G116C, which failed to induce any vesicle tethering. The other four proteins showed increased tethering rates and shorter lag times with Cer-containing vesicles (Fig. 3).

The proteins causing higher liposome aggregation, in terms of ΔA_400_ and tethering rates, were GABARAP G116C and GABARAPL1 G116C (Figs. 2 and 3a). With GABARAP G116C, in the presence of Cer, the aggregates seemed to exceed 400 nm in size, i.e. surpassing the Rayleigh limit, as the apparent absorbance decreased with time (Fig. 2, GABARAP).

LC3C G126C did not reach high ΔA_400_ values (Fig. 2), but showed almost no lag time with either of the lipid compositions (Fig. 3b). Correspondingly, it was also the protein showing fastest and most extensive lipidation (Fig. 1b, c).

Lowest vesicle tethering was induced by LC3A C17S G120C (Figs. 2 and 3a), which showed the longest lag times among the LC3/GABARAP proteins tested. Its low activity made the effect of Cer even more visible as the lag time was shortened from ≈ 17 to ≈ 10 min (Fig. 3b).

It has previously been suggested [9] that each LC3/GABARAP protein may need to reach a specific lipidation threshold value, different for each homolog, in order to induce vesicle tethering. Therefore, the absence of measurable liposome aggregation with LC3B G120C or GABARAPL2 C15S G116C (Fig. 2) could be explained if their lipidation thresholds were above 30%, i.e. above the values reached in the experiments (Fig. 1c).

Furthermore, although LC3A C17S G120C and GABARAPL1 G116C followed similar lipidation trends (Fig. 1), their ability to induce vesicle tethering varied, LC3A C17S G120C showing much less tethering than GABARAPL1 G116C (Figs. 2 and 3a). This reinforces the hypothesis of a lipidation threshold, which would be different for each of these proteins. According to the lag times (Fig. 3b), GABARAPL1 G116C would reach its threshold during the first minutes after protein addditon, while LC3A C17S G120C would take a longer time.

In turn, the Cer-related lag time shortening (Fig. 3b) could be due to the proteins reaching their lipidation threshold faster in the presence of Cer (Fig. 1b). In fact, there was an increased vesicle tethering rate with Cer (Fig. 3a) that correlated with shorter lag times (Fig. 3b). Note that Cer did not appreciably decrease the lag time of LC3C G126C-induced vesicle aggregation, probably because this time was already very short in the absence of Cer. Thus the chemical event of protein lipidation is a pre-condition for lipidated-protein-induced tethering, but each of these processes occur through different mechanisms and with different kinetics: lipidation is probably first-order with respect to lipid (molecules), while tethering (aggregation) is second-order with respect to lipid vesicles. This could explain how, in highly lipidated Atg8 proteins, i.e. richly protein-coated vesicles, as in the case of LC3C G126C, tethering could start very early, but also reach saturation soon, due to steric hindrance of the intervening LUV. (See below results with enzymatic lipidation of LC3C).

Ceramide dose-dependence of the observed increase in LC3/GABARAP-promoted vesicle tethering was tested with LC3A and GABARAPL1, using LUV composed of ePC: DOPE: PEmal: Cer (50-x:20:30:x), in which the mol % Cer concentration was x = 0, 5, 10 or 15. The results are shown in Supplementary Fig. 2. The rate of vesicle tethering, as seen by an increase in the slope of vesicle suspension turbidity (ΔA_400_) vs. time curves, increased with Cer concentration, and the lag time of the process decreased, more clearly in the case of GABARAPL1 (when the absolute change in turbidity was one order of magnitude higher than with LC3A).

Overall, these results imply that, even if individual variations occur among the different homologs, when Cer is part of the lipid composition these proteins do not only need less time to reach the onset of vesicle tethering, but they also do so at a faster rate once vesicle aggregation starts. In summary, Cer appears to potentiate LC3/GABARAP ability to promote vesicle tethering.

#### Enzymatic lipidation approach

The enzymatic lipidation procedure requires a LC3/GABARAP protein with Gly at its C terminus, ATG7, ATG3, MgCl_2_ and ATP. The lipid compositions used in this approach were ePC: DOPE (50:50) and ePC: DOPE: eCer (40:50:10).

This kind of experiment had demonstrated extensive lipidation of LC3B in a previous study, in which bilayer composition was PC: DOPE: bovine liver PI: NBD-PE (35/45/10/10 mol ratio) [8]. (Note that, in that early study, LC3B was called LC3). In the said study, LC3B was effective, though less than GABARAP, in promoting vesicle-vesicle fusion. LC3B was also notable in that, according to cryo-electron microscopy observations, it originated elongated vesicles, while GABARAP gave rise to large, spherical ones. Also in [8], LC3B exhibited very similar vesicle tethering properties irrespective of the chemical or enzymatic method of in vitro lipidation. Moreover, LC3B lipidation in vesicles composed of PC: DOPE: PI: DOG (33:55:10:2 mol ratio), and subsequent vesicle tethering and, to a small extent, lipid mixing was also observed in the presence of ATG7, ATG3, and the ATG12–ATG5-ATG16L1 (also known as E3) complex [9].

In the current study, just three of the six homologs, LC3A, GABARAP and GABARAPL1 were chosen for experiments following the enzymatic lipidation approach. GABARAP and GABARAPL1 were selected as the two proteins showing highest tethering effect with the chemical approach (Fig. 3a). LC3C was not included in this part of the study because previous experiments from this laboratory [11] indicated that its behavior, and probably its in vivo function too, differed from the other homologs, thus making it worthy of a separate investigation [30] (see below). LC3A was chosen as a representative of the LC3 subfamily.

**Fig. 4:**
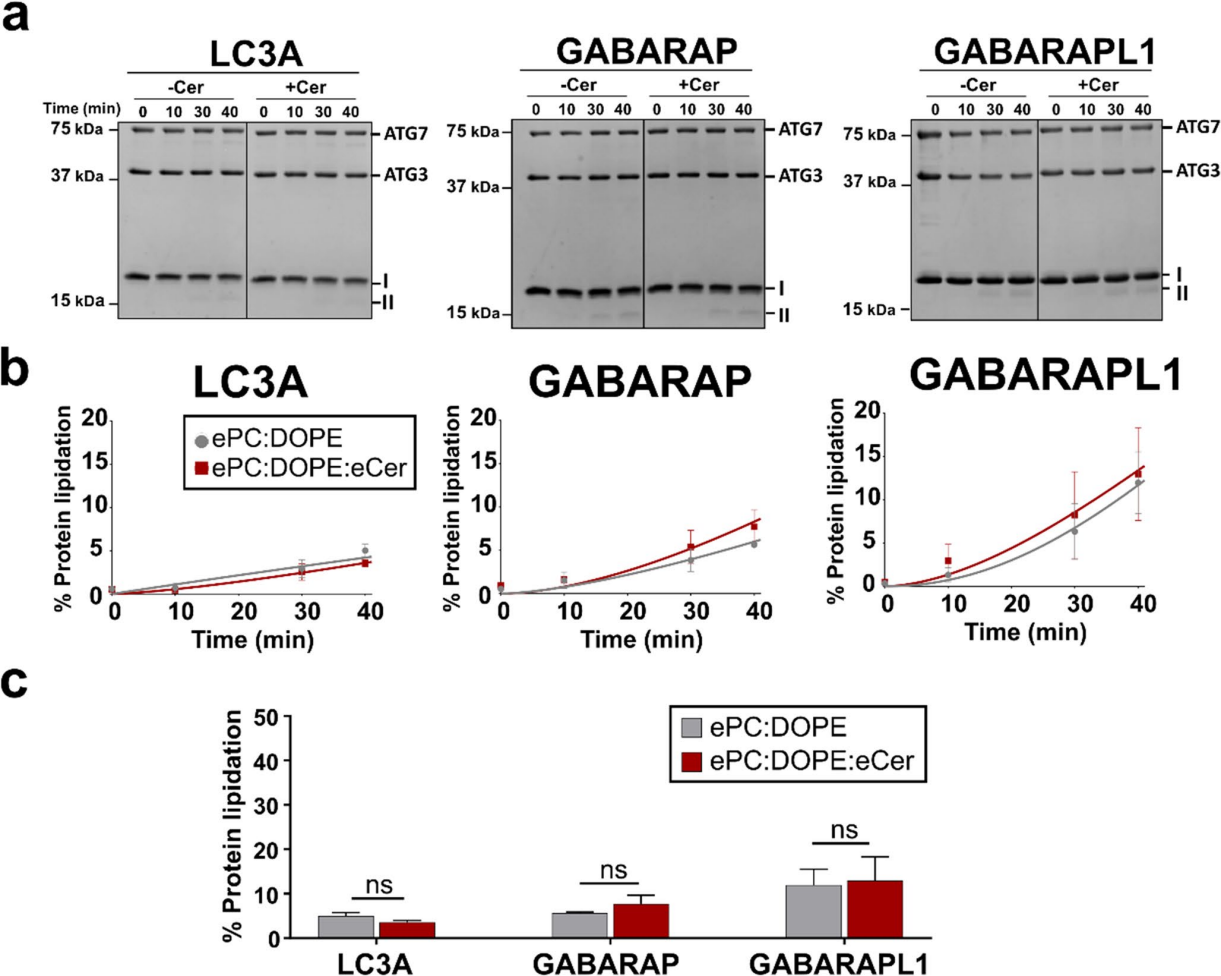
Ceramide does not enhance enzymatic lipidation of LC3A, GABARAP or GABARAPL1. Lipidation measured at 0, 10, 30 and 40 min after addition of 0.4 mM LUV, 0.5 µM ATG7, 2 µM ATG3, 1 mM MgCl_2_, 5 µM LC3/GABARAP, 5 mM ATP. (**a**) Representative SDS-PAGE gels for each protein and lipid composition. **-Cer** panels: ePC: DOPE (50:50); **+Cer** panels: ePC: DOPE: eCer (40:50:10). **I**, non-lipidated protein; **II**, lipidated protein. (**b**) Time course of protein lipidation. Data from experiments in Fig. 4a. (**c**) Percent protein lipidation at time 40 min. Data from experiments in Fig. 4b. For b andc c, LUV were composed of ePC: DOPE (50:50) (gray) or ePC: DOPE: eCer (40:50:10) (red). Liposomes were ≈ 80 nm in diameter. Average data ± S.D., *n* = 3. ns: non-significant differences

Lipidation levels of the three proteins (LC3A, GABARAP and GABARAPL1) were measured for 40 min. In all cases, lipidation values were much lower (< 20%) than those found with the chemical approach (Fig. 1 vs. Figure 4). A slight tendency towards a higher lipidation with Cer was observed with GABARAP and GABARAPL1, but differences measured at the end of the experiment were not significant (Fig. 4b, c). Under our conditions, at least for LC3A, no lipidation above 15–20% could be achieved with the enzymatic protocol even after very long reaction times (180 min) (data not shown).

**Fig. 5:**
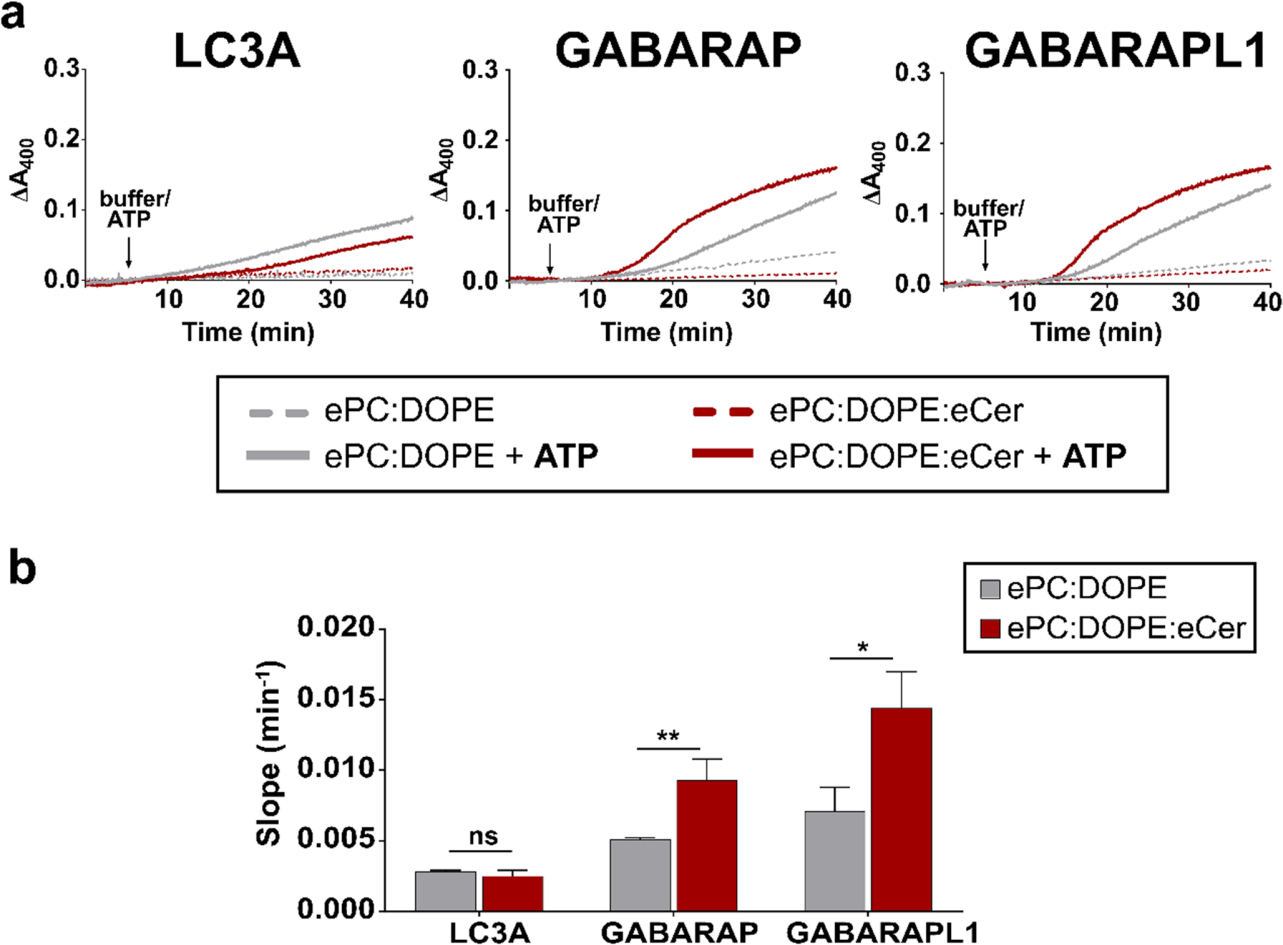
Ceramide increases the rate of liposome tethering induced by enzyme-lipidated GABARAP or GABARAPL1. Liposome tethering was assayed as ΔA_400_. 0.4 mM LUV composed of ePC: DOPE (50:50) (gray) or ePC: DOPE: eCer (40:50:10) (red) were mixed with 0.5 µM ATG7, 2 µM ATG3, 1 mM MgCl_2_, 5 µM LC3/GABARAP. Liposomes were ≈ 80 nm in diameter. (**a**) Vesicle tethering representative time courses. Arrows indicate 5 mM ATP (solid lines) or buffer (dashed lines) addition. (**b**) Vesicle tethering rates. Average data ± S.D., *n* = 3. **p < 0.01, *p < 0.05, ns: non-significant differences

Next, the vesicle tethering ability of each protein was tested (Fig. 5). In all cases, ATP addition had a positive effect: all three proteins were able to induce some vesicle aggregation (Fig. 5a, solid vs. dashed lines). GABARAP and GABARAPL1 exhibited a similar behavior: Cer almost doubled their vesicle tethering rates, GABARAPL1 reaching a higher rate (Fig. 5b). As expected, LC3A showed the smallest effect, with lower tethering rates and no Cer effects (Fig. 5b). This was expected as almost no lipidation was detected for this protein (Fig. 4c) and, according to the results obtained with the chemical approach, the lipidation threshold seemed to be higher for this protein. Note that, both for GABARAP and GABARAPL1, a very small ceramide-enhanced enzymatic lipidation (Fig. 4c) was accompanied by a sizable Cer-dependent increase in vesicle tethering rates (Fig. 5b and Supplementary Fig. 2). This may be an indication of different, complementary effects of Cer on protein lipidation and vesicle tethering. Note that lipidation and tethering are essentially different processes, in that each step of lipidation involves one protein molecule and one vesicle, ultimately a single Cer molecule, while the elementary event of tethering involves two vesicles, with the corresponding kinetic implications (see Discussion).

In summary, even if the enzymatic lipidation approach allowed a lower Cer effect on lipidation (Fig. 4c), the increasing effect on tethering rates was noticeable and significant for GABARAP and GABARAPL1 (Fig. 5b), prompting the study of Cer effects on their lipid mixing ability.

### Ceramide increased intervesicular lipid mixing induced by GABARAP and GABARAPL1

**Fig. 6:**
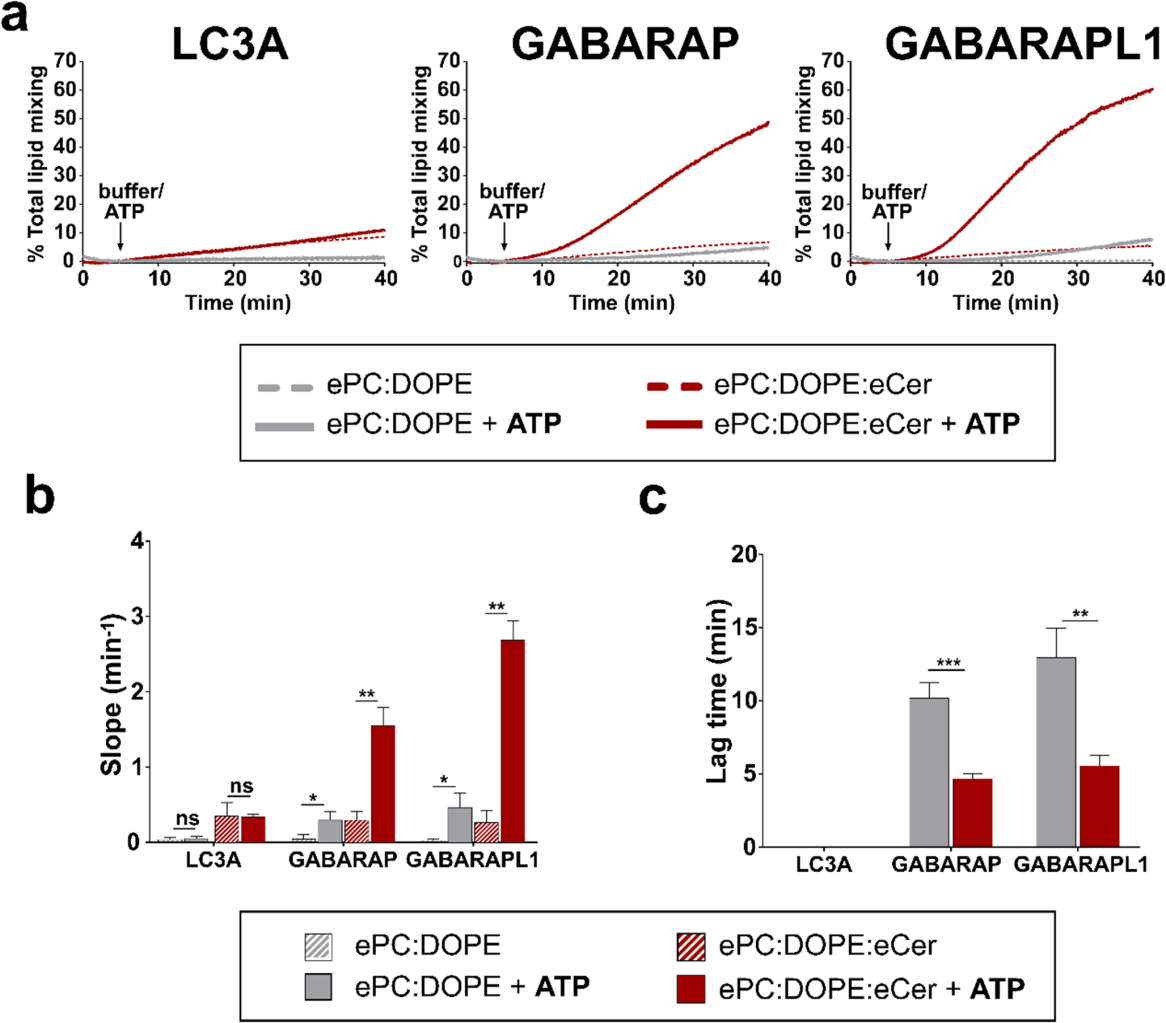
Both GABARAP and GABARAPL1 induce extensive lipid mixing of ceramide-containing vesicles. Total lipid mixing (TLM) induced by the lipidated proteins, monitored with the NBD-PE/Rho-PE lipid dilution assay. 0.4 mM LUV composed of ePC: DOPE (50:50) (gray) or ePC: DOPE: eCer (40:50:10) (red) were mixed with 0.5 µM ATG7, 2 µM ATG3, 1 mM MgCl_2_, 5 µM LC3/GABARAP protein. Liposomes were ≈ 80 nm in diameter. (**a**) Total lipid mixing, representative time courses. The arrows indicate 5 mM ATP (solid lines) or buffer (dashed lines) addition. (**b**) Total lipid mixing rates: -ATP (striped bars) or + ATP (solid bars). (**c**) Total lipid mixing lag times after ATP addition. (b, c) Data from experiments as in (a). Average data ± S.D., *n* = 3. ***p < 0.001, **p < 0.01, *p < 0.05, ns: non-significant differences

Liposome tethering is the initial step in the hemifusion or fusion between two vesicles. Any fusion event involves also lipid mixing between the fusing vesicles. Therefore, to test whether LC3/GABARAP were able to induce any kind of vesicle fusion, total lipid mixing (TLM) assays were performed (Fig. 6). For this approach, a vesicle population doped with the fluorophores NBD-PE and Rho-PE was mixed to a 1:9 ratio with unlabeled liposomes of the same lipid composition. NBD-to-Rho Förster energy transfer was measured.

The results showed that GABARAP and GABARAPL1 caused ATP-dependent, extensive lipid mixing only when the lipid composition included Cer (Fig. 6a, solid gray vs. red lines). Cer effect could be seen also in terms of a higher maximum initial slope (Fig. 6b, solid red bars) and of shorter lag times (Fig. 6c, solid red bars).

Even if some vesicle tethering was observed with LC3A (Fig. 5a), it was not associated to TLM under the assay conditions (Fig. 6a). No lag time could be detected either. With either lipid composition, the time courses after adding ATP overlapped with their respective ATP-free (non-lipidated) controls (Fig. 6a, dashed vs. solid red lines and Fig. 6b, stripped vs. solid red bars). This could indicate either that higher levels of lipidation are needed for LC3A or that this protein is unable to promote intervesicular lipid mixing.

Considering that LC3C was, together with GABARAP and GABARAPL1, the chemically lipidated protein giving rise to a higher extent of vesicle tethering (Fig. 2), experiments of tethering and TLM were performed with enzyme-lipidated LC3C. This protein caused robust vesicle tethering and TLM, and both events were clearly enhanced by Cer (Supplementary Fig. S3), even if Cer failed to improve lipidation, in agreement with the behavior of the other Atg8 proteins (Fig. 4).

Taken together, these results implied that the main Cer effect was to enhance lipid mixing, even without large increases in protein lipidation or vesicle tethering. Only when Cer was present, and the proteins could be lipidated (+ ATP), were they able to induce vesicle lipid mixing (Fig. 6).

### Ceramide enhanced GABARAP- and GABARAPL1-induced leakage-free inner-monolayer lipid mixing

**Fig. 7:**
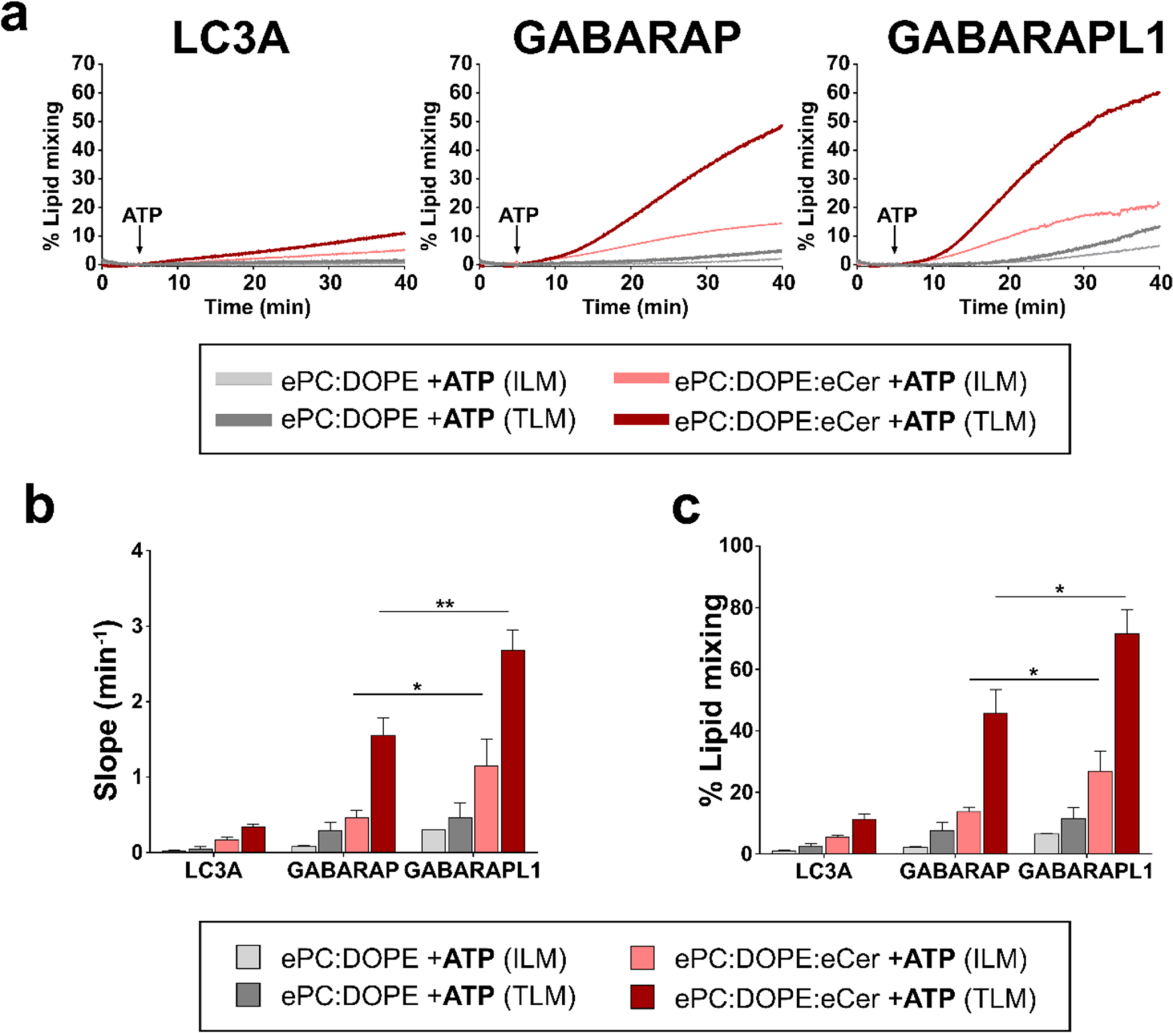
Both GABARAP and GABARAPL1 induce fusion in a fraction of the vesicle population. Total (TLM) and inner (ILM) lipid mixing induced by the lipidated proteins, monitored with NBD-PE/Rho-PE lipid dilution assays. 0.4 mM LUV composed of ePC: DOPE (50:50) (gray) or ePC: DOPE: eCer (40:50:10) (red) were mixed with 0.5 µM ATG7, 2 µM ATG3, 1 mM MgCl_2_, 5 µM LC3/GABARAP protein. Liposomes were ≈ 80 nm in diameter. Light colors ILM, dark colors TLM. (**a**) Representative time courses. The arrows indicate 5 mM ATP addition. (**b**) Total and inner lipid mixing rates. (**c**) Percent total and inner lipid mixing at 40 min. (b, c) Data from experiments as in (a). Average data ± S.D., *n* = 3, **p < 0.01, *p < 0.05

In full vesicle fusion, lipids from both monolayers of each membrane become mixed; in hemifusion, only the outer monolayer lipids exchange, while the inner monolayers do not come into contact. Since total lipid mixing assays (TLM) cannot differentiate between those two events, specific inner-monolayer lipid mixing assays (ILM) were performed, in combination with the above-mentioned TLM (Fig. 6), to distinguish which of the two events were taking place (Fig. 7).

With ePC:DOPE vesicles, neither GABARAP nor GABARAPL1 induced any marked ILM, the effect of GABARAP being virtually undetectable, and GABARAPL1 reaching only ≈ 10% after 35 min (Fig. 7a, light gray lines and bars). Both proteins were able to induce some ILM when Cer was part of the lipid mixture, although mixing never went beyond 30% (Fig. 7b, c). Therefore, GABARAP and GABARAPL1 seemed to have the capacity of inducing full fusion, but not of all the liposomes present. GABARAPL1 promoted faster and more extensive fusion than GABARAP.

The low fusogenic activity of LC3A, already hinted at in the previous sections (Figs. 5 and 6), was confirmed by the results in Fig. 7: with ePC: DOPE (50:50) no ILM was detected, and it barely reached 5% with ePC: DOPE: eCer after 35 min.

**Fig. 8:**
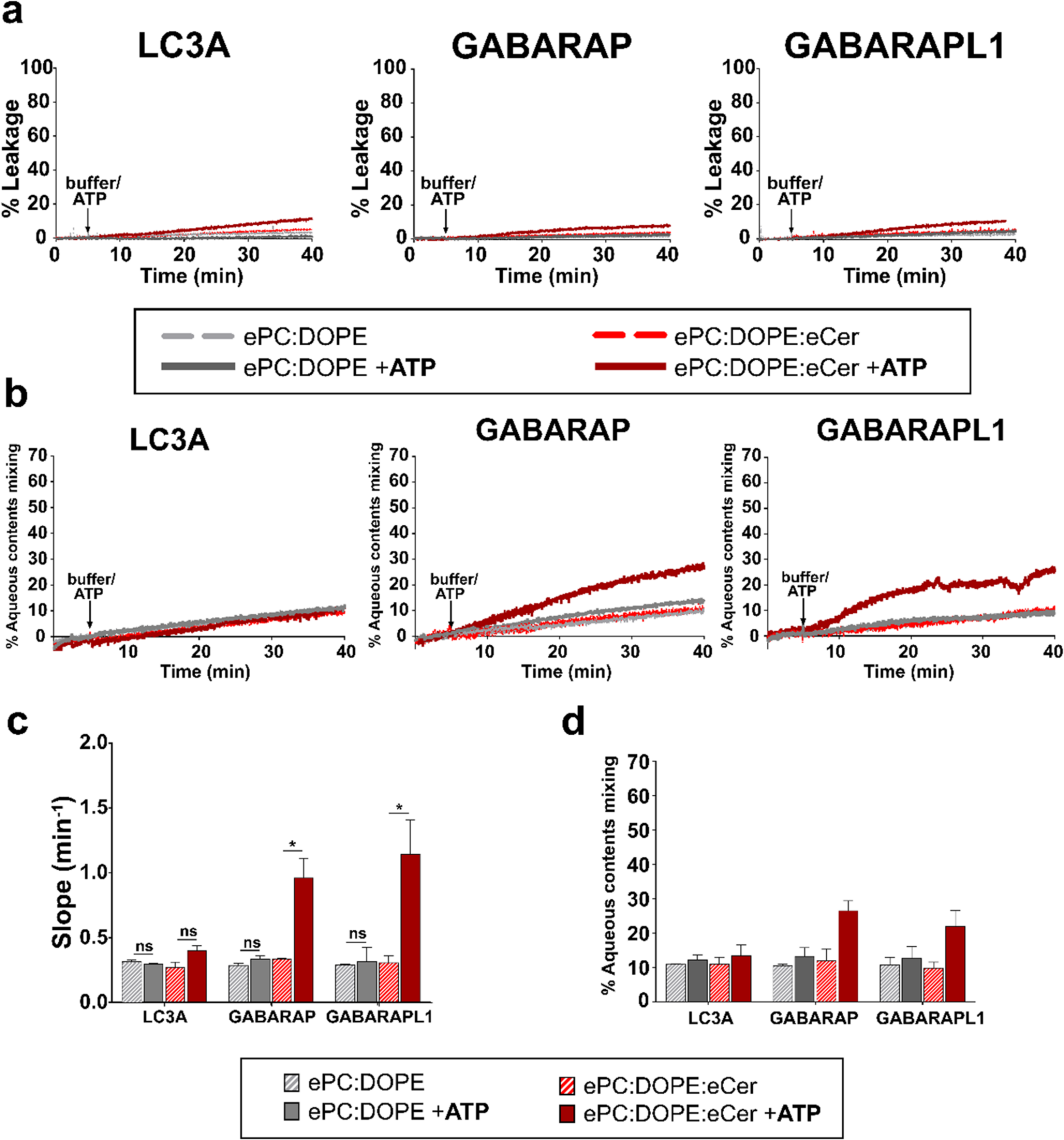
Both GABARAP and GABARAPL1 induce leakage-free intervesicular aqueous contents mixing. (**a**) Vesicle contents leakage induced by the lipidated proteins, monitored with an ANTS/DPX mixing assay. (**b**) Aqueous contents mixing (ACM) monitored with an ANTS/DPX mixing assay. (a, b) Representative time courses. The arrows indicate 5 mM ATP (solid lines) or buffer (dashed lines) addition. (**c**) Aqueous contents mixing rates. (**d**) Percent aqueous contents mixing at 40 min. (c, d) Data from experiments in (b). -ATP (striped bars) or + ATP (solid bars). Average data ± S.D., *n* = 3. ***p < 0.001, **p < 0.01, *p < 0.05, ns: non-significant differences. 0.4 mM LUV composed of ePC: DOPE (50:50) (gray) or ePC: DOPE: eCer (40:50:10) (red) were mixed with 0.5 µM ATG7, 2 µM ATG3, 1 mM MgCl_2_, 5 µM LC3/GABARAP protein. Liposomes were ≈ 80 nm in diameter. Contents leakage and mixing were assayed as detailed under Methods

An additional parameter to characterize the fusogenic effects of LC3/GABARAP is the mixing of vesicle aqueous contents (ACM). The assay involves testing whether vesicles containing a water-soluble fluorophore (ANTS) fuse with liposomes containing its quencher (DPX). Previously, it is essential to check that the decrease in ANTS fluorescence is not due to an increased membrane permeability (and to the subsequent contents leakage). Figure 8a shows that, neither in the absence nor in the presence of Cer, did any of the three LC3/GABARAP proteins induce a sizable vesicle permeabilization.

The lack of aqueous contents leakage allowed for reliable ACM assays in which LUV, containing ANTS or DPX, were mixed at a 1:1 ratio. Results in Fig. 8b-d supported what was previously observed in ILM assays (Fig. 7). With GABARAP or GABARAPL1 and ePC: DOPE vesicles, no ACM differences were observed in the absence or presence of lipidated proteins (Fig. 8b, dashed vs. solid gray lines; Fig. 8c, d, striped vs. solid gray bars). With liposomes composed of ePC: DOPE: eCer, ATP addition to GABARAP and GABARAPL1 induced a measurable ACM, significantly different from the controls (i.e. non-lipidated proteins) (Fig. 8b, dashed vs. solid red lines; Fig. 8c, d, stripped vs. solid red bars). In the case of LC3A, no significant differences were seen in any case. Therefore, these results reinforce the role of both Cer and the lipidation of GABARAP and GABARAPL1 (+ ATP) to enhance vesicle fusion (Fig. 8).

Taken together, these results support the notion that Cer promotes the fusion-inducing capacity of LC3/GABARAP proteins. Both the ILM and the ACM assays showed that GABARAP and GABARAPL1 proteins seem to have the capacity to induce vesicle hemifusion and that full fusion only happens in a fraction of the vesicles present in the assay mixture.

### Cer does not act through overall changes in membrane curvature

Two important Cer properties are that (a) it is a fusogenic lipid, and (b) it possesses a marked intrinsic negative curvature [18, 31]. Since PE is also known to facilitate membrane fusion, it is necessary to ascertain that the observed Cer effects are due to a specific property of this lipid, and not to a mere increase in the proportion of fusogenic lipids (PE + Cer) in the mixture. To this aim, two series of experiments were performed. One involved the use of ^31^P-NMR to detect possible non-lamellar (inverted hexagonal, cubic) phases in mixtures with Cer [32]. These would be an indication of a Cer-induced increase in negative curvature. Specifically, the following mixtures were examined, at 37 °C and 50 °C: ePC: DOPE (50:50); ePC: DOPE (40:60); ePC: DOPE: eCer (50:40:10), and ePC: DOPE: eCer (40:50:10) (mol ratios). At 37 °C, the temperature at which all the fusion experiments in this paper were performed, the spectral shape, with a shoulder at the low-frequency side, was characteristic of a lamellar phase [33]. No signal components suggestive of hexagonal or isotropic phases were detected, neither in the absence or in the presence of Cer (Supplementary Fig. S4). The same was true at 50 °C, a temperature at which formation of non-lamellar phases should be favored [32]. It can be concluded that, at the 10 mol% concentrations used in the present study, Cer does not induce any marked increase in the overall negative curvature of the bilayers.

A separate approach consisted of repeating the GABARAPL1-promoted lipid mixing experiments with ePC: DOPE (50:50), ePC: DOPE: eCer (50:40:10), and ePC: DOPE: eCer (40:50:10) mol ratios. The results showed (Supplementary Fig. S5) that Cer exerted a similar increase in lipid mixing when the negative-curvature/zero-curvature lipid ratio was either 1.0 or 1.5 (when the total negative-curvature lipids (DOPE + Cer) were either 50–60 mol% in the bilayer mixture). This speaks in favor of a specific fusogenic activity of Cer, unrelated to its negative curvature properties. Under the conditions of the experiment, Cer did not modify the extent of GABARAPL1 lipidation (Supplementary Fig. S5), in agreement with the above observations (Fig. 4), thus reinforcing the idea of a specific Cer ability to enhance vesicle-vesicle fusion.

## Discussion

Cer, usually present at low proportions in cellular membranes, has been shown to increase up to 10 mol% total lipid under different stress stimuli. It is known to act as a second messenger [34, 35]. Cer tends to create Cer-rich domains [36], modifying the fluidity and thickness of the membrane in those specific spots. Furthermore, Cer is known to promote membrane fusion [37, 38], mainly when it is generated by sphingomyelinases (SMases). This, together with recent work indicating the presence of sphingolipids in the phagophore membrane [13], as well as the putative Cer role in autophagy [17], made relevant our studies on phagophore expansion.

### Cer-enhanced vesicle tethering as shown by the chemical and enzymatic methods

The aim of this work was to investigate the potential role of Cer in the LC3/GABARAP-dependent incorporation of new lipid vesicles to the phagophore, in order to promote its expansion. Previous studies with knockouts of all six mammalian LC3/GABARAP homologs had found that autophagy could occur in the absence of those proteins [39]. However, in the latter cases, AP were formed at a much slower rate, they were smaller, and had often trouble fusing with lysosomes. The data point to an important, if not essential, role of LC3/GABARAP family in phagophore expansion [40]. Participation of LC3/GABARAP in that step is supported by their known ability to promote in vitro intervesicular lipid mixing once lipidated [8–11].

Recently, Bieber et al. [41]. studied the structural properties of the phagophore and the phagophore-organelle contact sites in yeast. They observed that the phagophore rim diameter was of ≈ 15 nm, and found vesicles < 100 nm in diameter in close contact with the phagophore. For this reason, the high-curvature vesicles (80–100 nm) used in this work could resemble those participating in the in vivo fusion events.

In vitro lipidation of LC3/GABARAP proteins was performed with either a chemical [8, 30] or an enzymatic method [8, 9]. The chemical method requires only the maleimide-modified lipid interaction with a protein Cys. This system is considered to accurately mimic the PE interaction with LC3/GABARAP [8, 11, 42]. Its simplicity allowed the comparison of the six LC3/GABARAP homologs in the absence of other autophagy proteins. The chemical approach showed that, when the bilayer lipid composition included 10 mol% Cer, vesicle-vesicle tethering rates were increased, and lag times were shortened, for most proteins (LC3A, LC3C, GABARAP and GABARAPL1) (Fig. 3). The proteins reaching lowest lipidation levels with this method, LC3B and GABARAPL2 (Fig. 1), were unable to induce vesicle tethering (Fig. 2). The higher affinity of LC3/GABARAP proteins for ceramide-containing vesicles, in the absence of PEmal, had been directly demonstrated by Varela et al. [29], using both monolayer and ultracentrifugation techniques.

Due to the similar structures of Ser and Cys (except that Ser lacks the thiol group) Ser was chosen to substitute the Cys residue in LC3A and GABARAPL2, so that the Cys-PEmal interaction could only happen through the protein C-terminal region. This mutation was already applied to GABARAPL2/GATE-16 in our previous work [8]. It could be argued that, in the chemical assay, the native proteins are modified. The use of C-terminal Cys instead of the native Gly would raise questions about the functionality and conformation of the protein. Additionally, the mutation of Cys to Ser to avoid unwanted Cys-maleimide interaction, along with the modification of 60 mol% of the original PE in the vesicles with a maleimide group (PEmal), could significantly alter the properties of the proteins and of the vesicles. Yet in our 2016 work [8] we demonstrated using several controls and experiments that these proteins, when lipidated with either the chemical or the enzymatic systems, worked in a very similar way. Moreover, Cys to Ser mutation was performed in that work with GABARAPL2 and it was found that it did not affect the structure or folding of that protein. As the overall structures of GABARAPL2 and LC3A are very similar, we consider that the Cys to Ser mutation does not affect the properties of the latter protein either.

Previous studies had suggested that each of the homologs might require a specific lipidation threshold in order to induce vesicle tethering [9]. In this regard, GABARAP and GABARAPL1 would be the proteins requiring lower lipidation thresholds. The results in Figs. 1, 2 and 3 support this hypothesis. This was especially relevant when comparing the chemically lipidated LC3A and GABARAPL1 (Figs. 1c and 3a), which showed similar lipidation levels although GABARAPL1 exhibited higher vesicle tethering.

Further evaluation of LC3A, GABARAP and GABARAPL1 with the enzymatic lipidation approach showed also differences among them (Fig. 5). Cer increased the vesicle tethering rates induced by GABARAP and GABARAPL1 (Fig. 5b), without changes in lipidation levels (Fig. 4), while for LC3A no Cer-attributable differences were seen. Apart from quantitative differences, both methods concurred in demonstrating that Cer enhanced vesicle tethering caused by GABARAP and GABARAPL1, and that LC3A was the protein exhibiting lowest effects among those three.

Two main (quantitative) differences were observed between the lipidations achieved with either the chemical or enzymatic methods, the chemical procedure leading to an overall more extensive Atg8 lipidation, and to a Cer-dependent increased lipidation, the latter Cer effect being almost absent with enzymatic lipidation. The origin of these differences would require a separate investigation, however, it has been shown [43] that PEmal, an N-maleimide-derivatized PE which substitutes to a large extent the native PE in the chemical lipidation assays, has the effect of increasing molecular order at the bilayer air-water interface. Such increased order could facilitate the essentially random “effective collisions” between Atg8 protein and lipid. Cer is also known to increase bilayer order [17, 18], thus it could as well cooperate in chemical lipidation.

The mechanism of enzymatic lipidation is unclear at present, but it is doubtful that it includes random collisions between PE and Atg8, since LC3 family members exist bound to ULK multiprotein complexes through the LIR domains in the latter [44]. Thus, enzyme catalysis based on facilitating the relative orientation of lipid and protein is more likely to predominate, and in this case bilayer order would be less important.

As shown above, enzymatic lipidation of Atg8 is not enhanced by Cer (Fig. 4), while, at least for GABARAP and GABARAPL1 (Fig. 5) and for LC3C (Supplementary Fig. S3), Cer bolsters vesicle-vesicle tethering. This is a probable indication that lipidation and tethering are mechanistically different events, even if one (lipidation) is a pre-requisite for the other (tethering). From a mechanistic point of view, lipidation consists of a chemically simple ligase reaction [45], while vesicle aggregation, or tethering, involves second- or higher-order physical-chemical processes in which several vesicles are brought to close contact through the lipidated Atg8 proteins, a process accompanied by the removal of many water molecules from the intervesicular space [9, 20]. When this whole panorama is considered, the partial divergence of Cer effects on lipidation and tethering becomes, if not clarified, at least understandable.

### Cer-enhanced vesicle fusion caused by GABARAP and GABARAPL1

Cer effect was most evident when the intervesicular lipid mixing ability of the proteins was assessed. With Cer-containing liposomes, LC3A had almost no effect as compared to the controls (no ATP addition), but GABARAP and GABARAPL1 generated a sizable amount of vesicle lipid mixing (Fig. 6). This extensive mixing was observed only when Cer was present in the lipid composition, in spite of the fact that those two proteins induced vesicle tethering even in the absence of Cer (Fig. 5). Moreover, both proteins displayed the ability to induce ILM (Fig. 7) and ACM (Fig. 8), suggesting that, once lipidated, they were capable of promoting full fusion or that, at least, a fraction of the vesicles in the assay could. Cer effects on protein lipidation, vesicle tethering and vesicle-vesicle fusion (lipid mixing + aqueous contents mixing) are jointly shown in the Supplementary Figure S6, to facilitate comparison. Our previous results [9, 29] showed that, with cardiolipin-containing vesicles, Cer promoted membrane binding of pure soluble GABARAP (as well as the other LC3/GABARAP members). However, even in the case that the soluble protein would not bind Cer by itself, it cannot be excluded that the lipidated (PE-bound) protein could do so, playing a direct role in hemifusion [13]. Our previous studies on vesicle-vesicle fusion, induced by phospholipase activities [32, 46, 47], in agreement with data from other authors [19], have shown a causal chain of effects from the phospholipase trigger to vesicle aggregation (tethering), hemifusion, and fusion, the latter demonstrated through intervesicular lipid mixing and intervesicular aqueous contents mixing, with or without contents leakage [48]. Once the fusogen (e.g. diacylglycerol) is formed in the bilayer, the chain of phenomena occurs without further intervention, and in a non-stop way. The kinetics of the various events may vary substantially from one to the next, but the order of events is invariable. In the present case, lipidation of a LC3/GABARAP protein constitutes the trigger of a chain of cause-effect phenomena, ultimately leading to vesicle-vesicle fusion.

In addition to LC3/GABARAP, other autophagy-related proteins could be required to complete the full fusion of new vesicles in vivo. Furthermore, this mechanism might work together with the direct lipid transport mediated by ATG2 and ATG9 [7, 49, 50]. Both vesicle-vesicle fusion and lipid transfer have been proposed to act in a concerted way, to achieve phagophore expansion and AP formation [41]. Moreover, the interaction between ATG2 and GABARAP, or GABARAPL1, has been described [51], suggesting some kind of interplay linking both systems.

Conically shaped lipids [18, 52, 53], of which ceramide is an example [54], are known to facilitate membrane fusion. However, the controls in which ATP was not added prove that lipid mixing specifically requires lipidation of GABARAP and GABARAPL1; Cer alone was not enough to generate the measured levels of lipid mixing (Fig. 6, dashed red lines). It is currently unknown whether the essential elements of AP expansion through fusion involve protein-protein, or protein-lipid, and/or other kinds of interaction [10, 40, 55–57]. Moreover, it has been proposed that LC3/GABARAP oligomerization would help bring the liposomes closer to each other [57, 58], but the way in which LC3/GABARAP mediate vesicle tethering and fusion is still not fully understood.

The differences between the ability of LC3A, GABARAP and GABARAPL1 in inducing intervesicular fusion are in agreement with the results obtained by Iriondo et al. [9]. with a lipid composition that included 10 mol% phosphatidylinositol (PI) and 2 mol% dioleoylglycerol (DOG). Those results supported the idea of different LC3/GABARAP proteins playing different roles in autophagy [9, 10, 59]. The above new results with 10 mol% Cer suggest that GABARAP and GABARAPL1 could be two LC3/GABARAP proteins participating in phagophore expansion, while LC3A would mainly play other roles in autophagy, such as cargo recognition [59]. Moreover, taking into account the results with GABARAP and GABARAPL1, and since those two proteins have already been suggested to bind the phagophore edges [9], the presence of Cer in those edges could favor and accelerate the process of vesicle fusion to the phagophore [29].

These studies have focused on the lipid-protein interactions of LC3/GABARAP proteins during phagophore expansion, but they may interact with Cer in other steps of the autophagic process or even in other organelles. As for autophagy, aSMases are found inside the lysosomes, where the produced Cer could interact with lipidated LC3/GABARAP proteins after AP-lysosome fusion, or before fusion if Cer was exposed in the outer leaflet of the lysosomal membrane. Furthermore, LC3/GABARAP proteins exhibit additional functions not directly related to the autophagy process, such as extracellular vesicle transport [60]. In this secretory pathway, not only LC3/GABARAP lipidation was observed, but also nSMase2, which produces Cer, was required.

In summary, our in vitro studies have demonstrated that (a) Cer exerts a direct effect on LC3/GABARAP-induced vesicle-vesicle tethering, independent from ATG3 or ATG7, and (b) Cer enhances vesicle fusion caused by GABARAP and GABARAPL1. (c) The data could shed light on fusion events happening in other cellular processes not necessarily involving autophagy.

## Supplementary Information

Below is the link to the electronic supplementary material.

Supplementary file1 (DOCX 1.11 MB)

## Data Availability

Data and materials will be made available upon reasonable request.

## Abbreviations

ACM: Aqueous contents mixing
ANTS: 8-Aminonaphthalene-1,3,6-trisulfonic acid, disodium salt
AP: Autophagosome
Atg8: Autophagy-related 8
ATG: Autophagy-related protein/s
ATP: Adenosine triphosphate
Cer: Ceramide/s
DOPE: 1,2-Dioleoyl-*sn*-glycero-3-phosphatidylethanolamine
DPX: p-Xylene-bis-pyridinium bromide
DTT: DL-dithiothreitol
ePC: L-α-phosphatidylcholine from hen egg yolk
GABARAP: GABA type A receptor-associated protein
GABARAPL1: GABA type A receptor-associated protein-like 1
GABARAPL2: GABA type A receptor-associated protein-like 2
ILM: Inner-monolayer lipid mixing
LUV: Large unilamellar vesicle/s
MAP1LC3A/LC3A: Microtubule-associated protein 1 light chain 3 alpha
MAP1LC3B/LC3B: Microtubule-associated protein 1 light chain 3 beta
MAP1LC3C/LC3C: Microtubule-associated protein 1 light chain 3 gamma
NBD-PE: N-(7-nitrobenz-2-oxa-1,3-diazol-4-yl)−1,2-dihexadecanoyl-*sn*-glycero-3-phosphatidylethanolamine
PE: Phosphatidylethanolamine
Rho-PE: 1,2-Dioleoyl-*sn*-glycero-3-phosphatidylethanolamine-N-lissamine rhodamine B sulfonyl
SL: Sphingolipid/s
SM: Sphingomyelin
TLM: Intervesicular total lipid mixing

## Declarations

### Research involving human and animal participants

No human subjects have been used.
