# Supplementary material for: Ceramide and the membrane-fusion activity of LC3/GABARAP autophagy proteins
Source: Cell Mol Life Sci. 2025 Jul 19;82(1):283. doi: 10.1007/s00018-025-05811-9 (PMC12276180; doi:10.1007/s00018-025-05811-9)
Supplement: Supplementary file 1 — Supplementary file1 (DOCX 1.11 MB) [file 18_2025_5811_MOESM1_ESM.docx]

**SUPPLEMENTARY FIGURES**

**
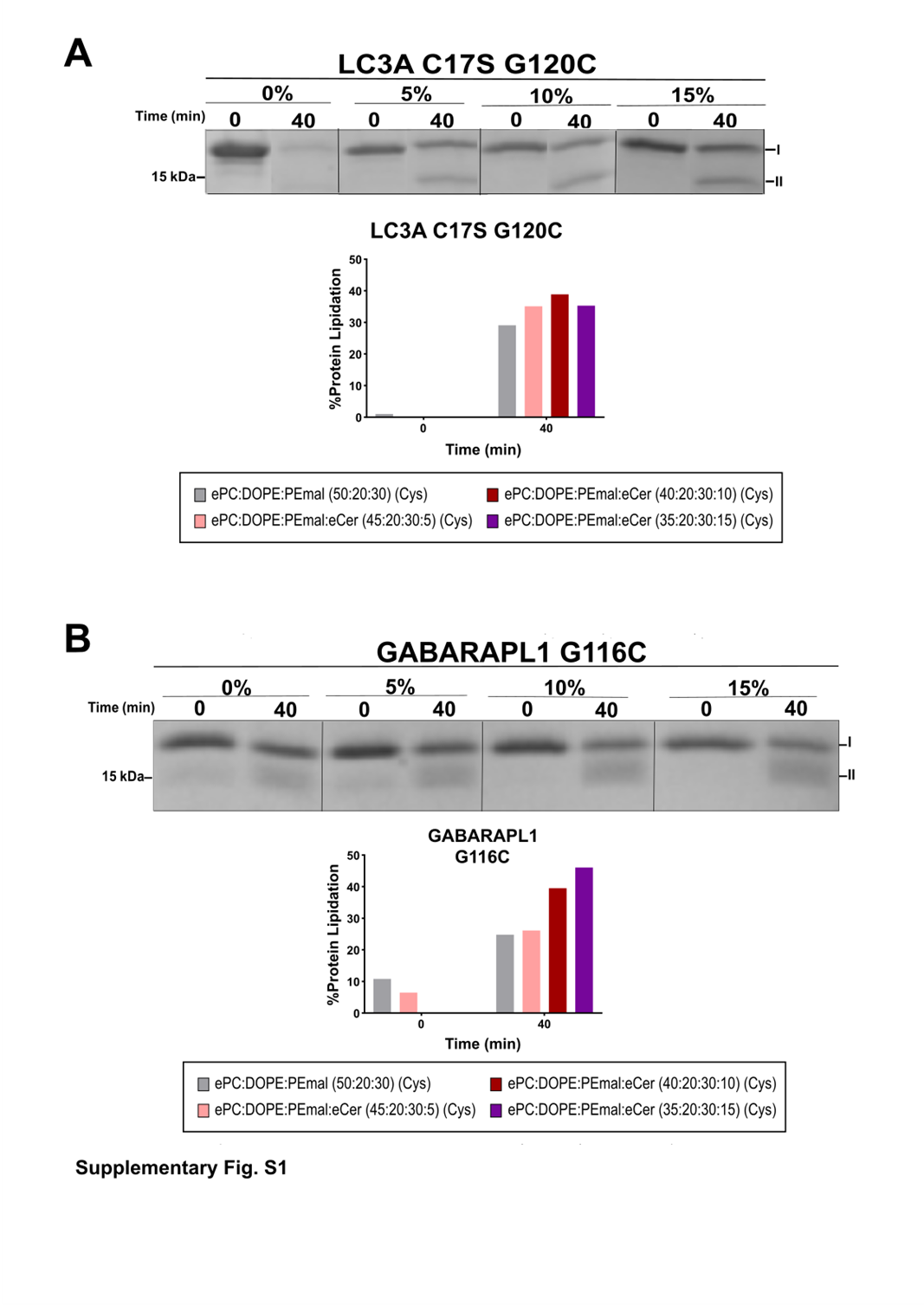
**

**Supplementary Figure S1. Ceramide dose-dependence of (A) LC3A and (B) GABARAPL1 chemical lipidation.** Protein Cys-terminal lipidation measured at times 0, and 40 min. LUV were composed of ePC:DOPE:PEmal:Cer (50-x:20:30:x), in which the mol % Cer concentration was x = 0, 5, 10 or 15. Representative gels are shown.

**
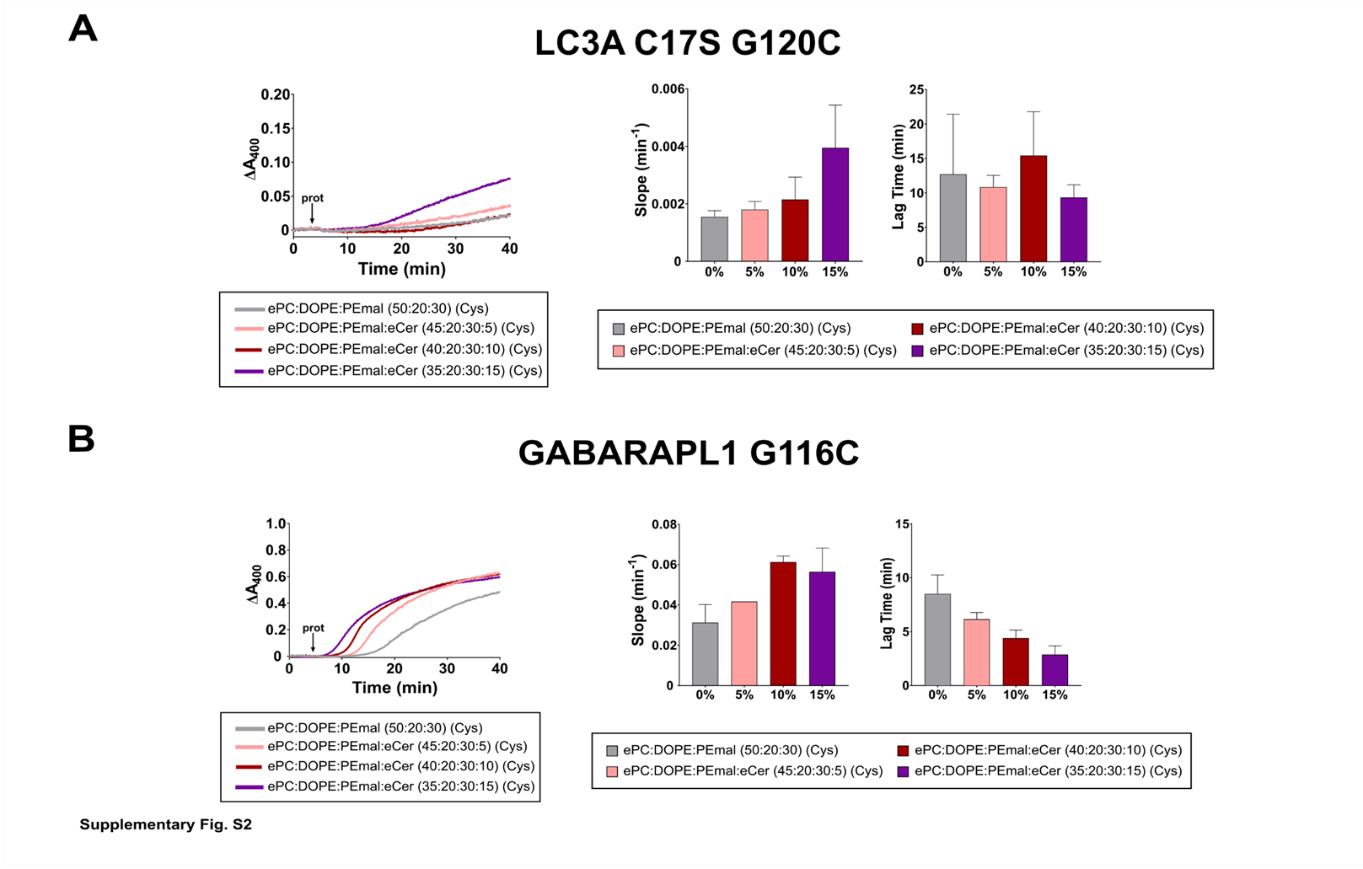
**

**Supplementary Fig. S2. Ceramide dose-dependence of the observed increase in LC3/GABARAP-promoted vesicle tethering, tested with (A) LC3A and (B) GABARAPL1.** Tethering was assessed through an increase in vesicle suspension turbidity (ΔA_400_ ). 0.4 mM LUV were used, composed of ePC:DOPE:PEmal:Cer (50-x:20:30:x), in which the mol % Cer concentration was x = 0, 5, 10 or 15. Representative curves are shown.

**
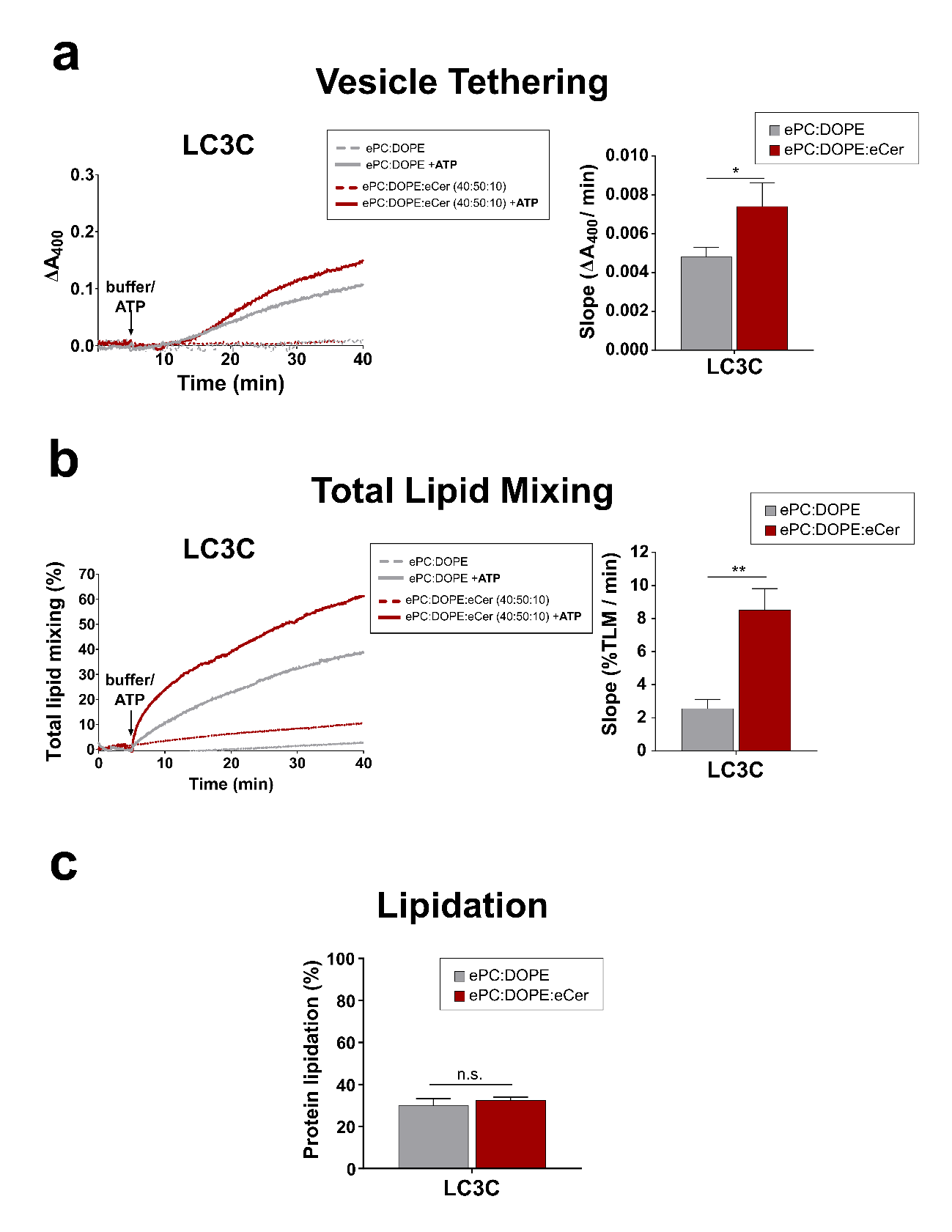
**

**Supplementary Figure S3. Ceramide increases the rate of liposome tethering and intervesicular lipid mixing induced by enzyme-lipidated LC3C.** (A) Vesicle tethering (aggregation). Left: representative time courses; right: tethering rates (slopes). Liposome tethering was assayed as ΔA400. (B) Intervesicular lipid mixing. Left: representative time courses; right: lipid mixing rates (slopes). Total lipid mixing (TLM) induced by the lipidated LC3C, was monitored with the NBD-PE/Rho-PE lipid dilution assay. (C) Percent protein lipidation 40 min after ATP addition. 0.4 mM LUV composed of ePC:DOPE (50:50) (gray) or ePC:DOPE:eCer (40:50:10) (red) were mixed with 0.5 μM ATG7, 2 μM ATG3, 1 mM MgCl_2_, 5 μM LC3C. Liposomes were ≈80 nm in diameter. Arrows indicate 5 mM ATP (solid lines) or buffer (dashed lines) addition. Average data ± S.D., n = 3. **p < 0.01, *p < 0.05, n.s.: non-significant differences.

**Supplementary Figure S4. Representative ^31^P-NMR spectra of lipid dispersions in buffer.** Lipids were in the form of multilamellar vesicles. Bilayer composition is indicated on top of the corresponding spectra. Top spectra: 50 °C. Bottom spectra: 37°C. In all cases, the spectral shape is compatible with phospholipids dispersed in bilayer (lamellar) form.

**
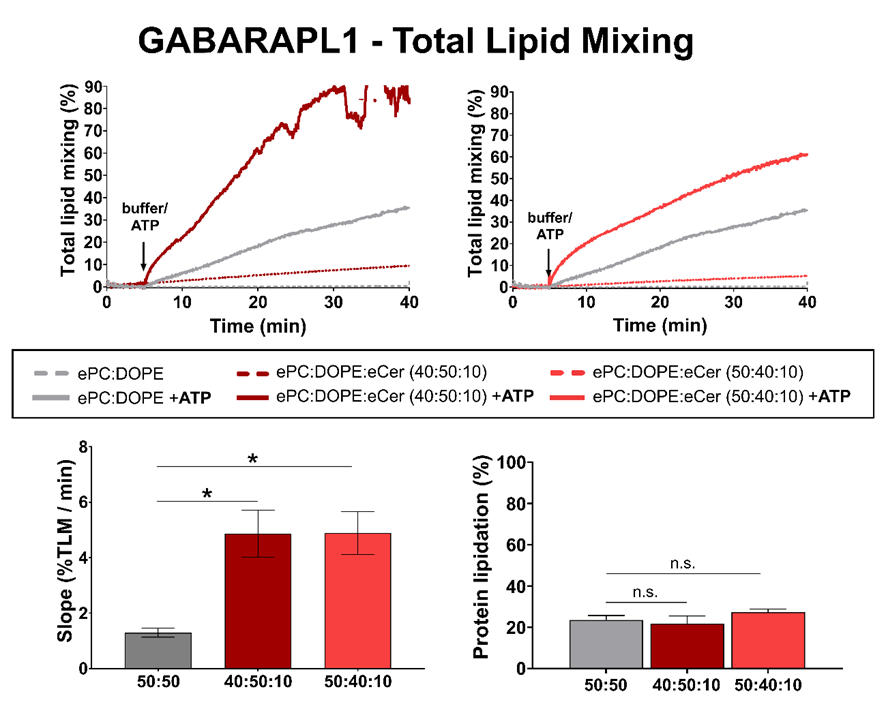
**

**Supplementary Figure S5. A comparison of GABARAPL1-induced intervesicular lipid mixing in LUV composed of ePC:DOPE (50:50) (gray), or ePC:DOPE:eCer (40:50:10) (dark red), or ePC:DOPE:eCer (50:40:10) (light red).** Top left: ePC:DOPE:eCer (40:50:10) representative time courses; top right: ePC:DOPE:eCer (50:40:10) representative time courses. Bottom left: Lipid mixing rates (slopes). Total lipid mixing (TLM) induced by the lipidated GABARAPL1 in the presence of 5 mM ATP, was monitored with the NBD-PE/Rho-PE lipid dilution assay. Bottom right: Percent GABARAPL1 lipidation 40 min after ATP addition. 0.4 mM LUV composed of ePC:DOPE (50:50) (gray) or ePC:DOPE:eCer (40:50:10) (dark red), or ePC:DOPE:eCer (50:40:10) (light red) were mixed with 0.5 μM ATG7, 2 μM ATG3, 1 mM MgCl_2_, 5 μM LC3C. Liposomes were ≈80 nm in diameter. Arrows indicate 5 mM ATP (solid lines) or buffer (dashed lines) addition. Average data ± S.D., n = 3. Student’s t-test: *p < 0.05, n.s.: non-significant differences.


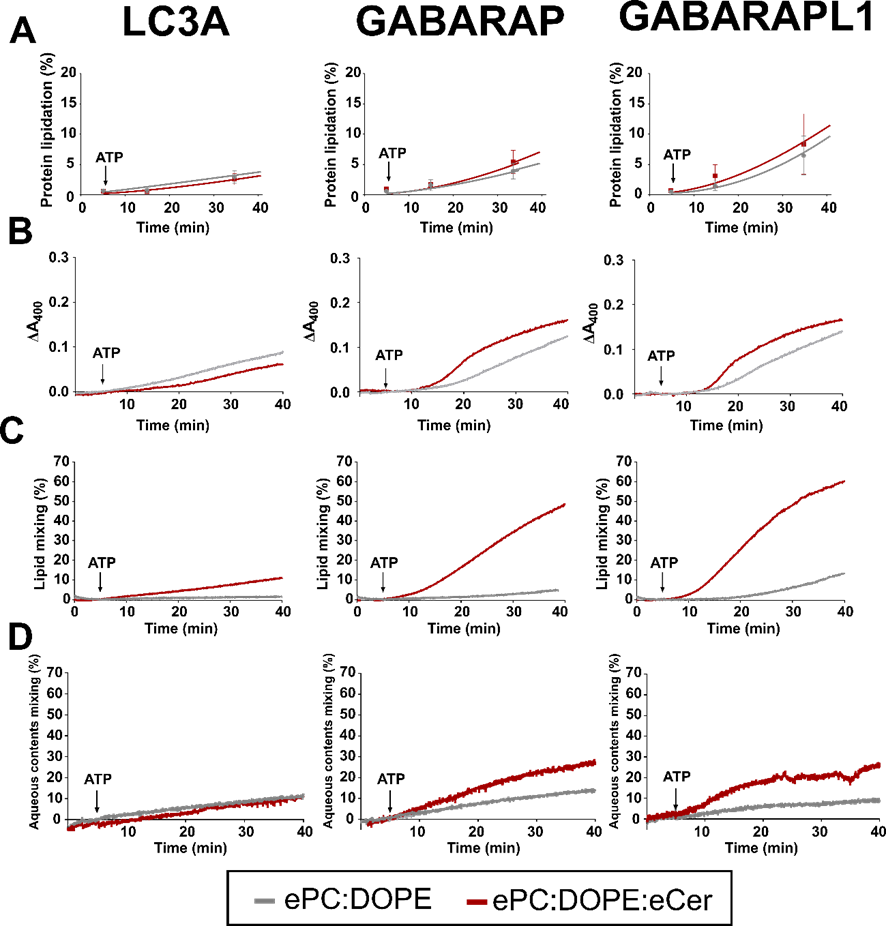


**Supplementary Figure S6. A summary of LC3/GABARAP-protein enzymatic lipidation, vesicle tethering, inter vesicular lipid mixing and aqueous contents mixing in the presence or absence of Cer.** Data from Figures 4, 5, 6, 8. Arrows indicate 5 mM ATP addition in all cases, in order to allow an easier comparison of the results in this study, in the absence (grey lines) or presence (red lines) of Cer. (A) LC3/GABARAP protein lipidation. (B) LC3/GABARAP-induced vesicle tethering. (C) Time courses of TLM. (D) Time courses of ACM. Representative time courses of three independent experiments.
